# IFI16 is required for DNA sensing in human macrophages by promoting production and function of cGAMP

**DOI:** 10.1038/ncomms14391

**Published:** 2017-02-10

**Authors:** K. L. Jønsson, A. Laustsen, C. Krapp, K. A. Skipper, K. Thavachelvam, D. Hotter, J. H. Egedal, M. Kjolby, P. Mohammadi, T. Prabakaran, L. K. Sørensen, C. Sun, S. B. Jensen, C. K. Holm, R. J. Lebbink, M. Johannsen, M. Nyegaard, J. G. Mikkelsen, F. Kirchhoff, S. R. Paludan, M. R. Jakobsen

**Affiliations:** 1 Department of Biomedicine, Aarhus University, Wilhelm Meyers Alle 4, Aarhus C 8000, Denmark; 2 Aarhus Research Centre of Innate Immunology, Aarhus University, Wilhelm Meyers Alle 4, Aarhus C 8000, Denmark; 3 Ulm University Medical Center, Institute of Molecular Virology, Meyerhofstr. 1, Ulm 89081, Germany; 4 The Danish Diabetes Academy supported by the Novo Nordisk Foundation, Aarhus University, Nordre Ringgade 1, Aarhus C 8000, Denmark; 5 New York Genome Center, 101 Avenue of the Americas, New York City, New York 10013, USA; 6 Department of Systems Biology, Columbia University, 1130 St Nicholas Avenue, New York City, New York 10032, USA; 7 Department of Forensic Medicine, Aarhus University Hospital, Palle Juul-Jensens Boulevard 99, Entrance 1, Aarhus N 8200, Denmark; 8 University Medical Center Utrecht, Heidelberglaan 100, Utrecht 3584 CX, Netherlands

## Abstract

Innate immune activation by macrophages is an essential part of host defence against infection. Cytosolic recognition of microbial DNA in macrophages leads to induction of interferons and cytokines through activation of cyclic GMP-AMP synthase (cGAS) and stimulator of interferon genes (STING). Other host factors, including interferon-gamma inducible factor 16 (IFI16), have been proposed to contribute to immune activation by DNA. However, their relation to the cGAS-STING pathway is not clear. Here, we show that IFI16 functions in the cGAS-STING pathway on two distinct levels. Depletion of IFI16 in macrophages impairs cGAMP production on DNA stimulation, whereas overexpression of IFI16 amplifies the function of cGAS. Furthermore, IFI16 is vital for the downstream signalling stimulated by cGAMP, facilitating recruitment and activation of TANK-binding kinase 1 in STING complex. Collectively, our results suggest that IFI16 is essential for efficient sensing and signalling upon DNA challenge in macrophages to promote interferons and antiviral responses.

## Abstract (web summary)

The role of IFI16 as a DNA sensor is highly controversial. With support from a Nature Communications back-to-back publication from Almine *et al*. the authors here provide functional evidence that IFI16 is required for DNA sensing via the cGAS-STING pathway in human macrophages.

Innate immune activation by cytosolic DNA from microbial pathogens is a potent trigger of type I interferon (IFN) and pro-inflammatory cytokines^1^. Interferon activation has been extensively studied both in terms of the proteins binding cytosolic DNA and those needed for subsequent downstream signalling and immune activation. Although many candidate sensors of cytosolic DNA have been suggested^2^, two proteins have been demonstrated by separate laboratories to play a role in DNA-driven type 1 interferon responses. These proteins are cyclic GMP-AMP synthetase (cGAS) and interferon gamma-inducible factor 16 (IFI16) (ref. 3). IFI16, a cytosolic and nuclear protein, is associated with induction of IFN-α and IFN-β on stimulation with single-stranded and double-stranded DNA^4^,^5^,^6^ and by infection with different herpesviruses^7^,^8^,^9^, human immunodeficiency virus type 1 (HIV-1)^5^ and bacterial infections such as Listeria and Francisella^10^,^11^. The cytosolic protein cGAS is important for sensing all forms of structured DNA, and is a pivotal sensor of microbial DNA^12^,^13^,^14^,^15^. cGAS has the enzymatic capacity to produce the second messenger cyclic GMP-AMP (cGAMP)^13^,^16^,^17^,^18^, which docks onto the endoplasmic reticulum-bound protein stimulator of interferon genes (STING). This interaction induces conformational changes that allow STING to homodimerize, migrate from the ER (ref. 19), and recruit TANK-binding kinase 1 (TBK1)^20^. How TBK1 is actively recruited to STING is currently unknown, but the absence of TBK1 binding to STING results in impaired immune activation^21^. A recent report demonstrated that TBK1 binding to STING initiates a complex cascade of events including phosphorylation of STING as well as recruitment and activation of interferon regulatory factor 3 (IRF3)^21^. Lack of phosphorylation of STING at Ser^366^ abolishes downstream signalling and immune activation, demonstrating the importance of precise and direct activation of STING.

Studies of cGAS-deficient mice show a clear phenotype in innate immune responses^13^,^22^,^23^. As mice do not have a direct ortholog to human IFI16, data from IFI16-deficient mouse models are not available. Many p200 family members have been suggested to have functions partly overlapping with human IFI16 (refs 24, 25). However, due to the lack of a definitive murine IFI16 ortholog, mouse models are not suitable to resolve the potential interconnection between cGAS and IFI16 in the innate immune response to foreign DNA.

In contrast to the well-described mechanism of action of cGAS in DNA sensing, there is limited knowledge regarding the interaction of IFI16 and STING-dependent signalling and also whether IFI16 is redundant to the cGAS-STING-TBK1 pathway. Previous findings have shown that the affinity of cGAS for DNA varies between relatively weak (Kd in the 20 μM range)^26^,^27^ to strong (≈80 nM)^28^ and that specific sizes or structures of the dsDNA are required for cGAS to engage binding^29^,^30^. Furthermore, cGAS binding to ssDNA is ineffective^28^. Thus, it seems plausible that cGAS responds efficiently to cytosolic DNA with help from one or more co-factors. As IFI16 can bind strongly to single and double-stranded DNA through its HIN domains and modulate protein-protein interactions via its PYRIN domain^5^,^31^,^32^, here we explored the mechanism by which IFI16 promotes DNA-driven STING-dependent signalling.

We present two functions of human IFI16 in the cGAS-STING pathway. Using human phorbol myristate acetate (PMA) treated THP1 cells and human monocyte-derived macrophages (MDMs) depleted of IFI16, we find that early interferon expression in the response to viral infections or DNA transfection requires IFI16. Importantly, in IFI16-deficient cells stimulated with DNA, the level of STING dimerization, phosphorylation and downstream signaling is compromized. Moreover, IFI16 is necessary for efficient cGAMP production through cGAS in response to DNA. Finally, IFI16 actively recruits TBK1 to the cGAMP-stimulated STING complex and thus promotes phosphorylation of STING. Collectively, our results suggest that IFI16 regulates STING activation and is an integrated part of the DNA sensing pathway in human macrophages.

## Results

### IFI16 controls innate immune responses to viral infections

**Figure 1:**
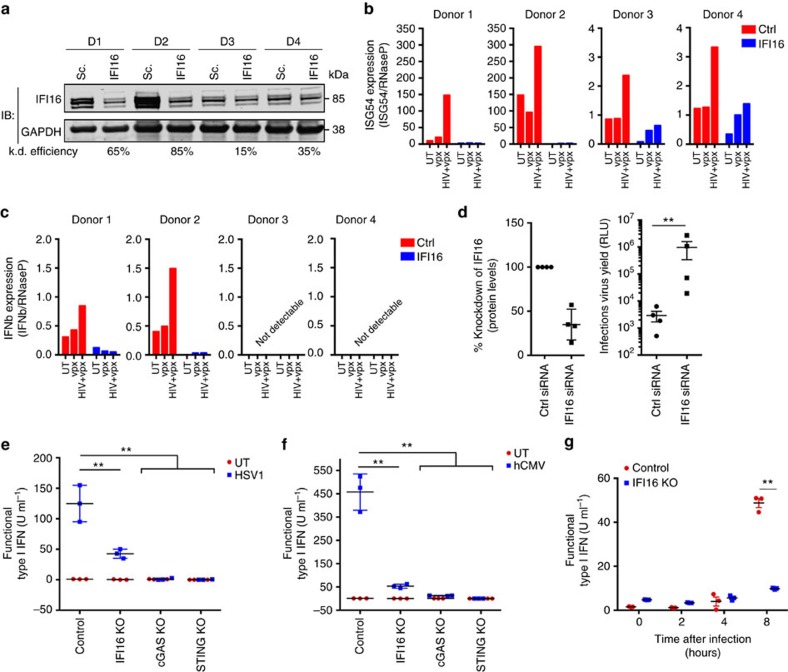
The immune response to HIV and HSV infection in human macrophages is regulated by IFI16. (**a**) IFI16 expression was measured by western blotting in four primary human macrophage donors treated with scrambled (Sc.) and IFI16-specific (IFI16) siRNA. (**b**,**c**) *ISG54* expression (**b**) and *IFN-β* expression (**c**) was measured in the four donors portrayed in (**a**) challenged with either Vpx particles alone or HIV^vpx+^ for 18 h. Data represent mRNA expression of each gene normalized to mRNA expression of *RNaseP* from two biological replicates of each donor. (**d**) IFI16 expression measured by WB in four primary human macrophages treated with scramble or IFI16-specific siRNA at the time of challenge with HIV-Bal infection (left panel). Production of replication competent HIV particles was measured 6 days p.i. using the HIV permissive TzmBL Luciferase reporter cell line (right panel). (**e**,**f**) Type I interferon expression was evaluated in control, IFI16 KO, cGAS KO and STING KO THP-1 cells challenged with HSV1 (**e**) or hCMV (**f**) 18 h after infection using a MOI of 3. (**g**) Type I interferon expression was evaluated in control and IFI16 KO cells at 2, 4 and 8 h after HSV1 infection using a MOI of 10. Data in (**e**–**g**) represent the mean±s.d. of biological triplicates, representative of two independent experiments. Unpaired *t*-test corrected for multiple comparisons using Holm–Sidak was been performed to evaluate the significance. *P<0.05; **P<0.01.

Recent reports have demonstrated that HIV reverse transcription intermediates (RTIs) can be detected by cGAS and initiate the canonical STING-interferon pathway in myeloid dendritic cells treated with the viral SAMHD1 antagonist Vpx (refs 12, 30, 33, 34). In macrophages, SAMHD1 may regulate both the pool of dNTPs, as well as direct degradation of HIV RNA^35^,^36^. Thus, degradation of SAMHD1 by Vpx enables higher production of HIV RTIs in the cytosol and thus more PAMPs for potential sensing. We therefore sought to determine whether IFI16 is involved in innate immune activation in primary human MDMs infected with replication competent HIV in combination with lentiviral particles packed with Vpx proteins (hereafter HIV^+vpx^). Using RNAi depletion of IFI16 in MDMs from four different donors, we achieved variable knockdown efficiencies ranging from 15 to 85% (Fig. 1a). Most efficient knockdown was achieved in the two donors showing the highest basal levels of IFI16 expression (Fig. 1a). Interestingly, the basal level of IFI16 correlated with induction of *ISG54* following infection with HIV^+vpx^. Donors 1 and 2 demonstrated robust *ISG54* induction in scrambled siRNA-treated MDMs (Fig. 1b; range 150–300 fold). By contrast, donors 3 and 4 presented a modest increase in *ISG54* expression (Fig. 1b: range 2–3 fold increase) when infected with HIV^+vpx^. Vpx alone had no significant effect. Similar observations were made for *IFN-β* expression (Fig. 1c). However, here we only detected a signal above background for donors 1 and 2, in which case infection with HIV^+vpx^ demonstrated clear *IFN-β* induction. Of note, the poor response to HIV infection observed for donor 3 was not because of reduced basal expression of cGAS or STING (Supplementary Fig. 1a). More importantly, MDMs treated with IFI16-specific siRNAs did not show an induction of *ISG54* and *IFN-β* expression after HIV infection (Fig. 1b,c). To confirm that these results were not due to unspecific effects of the siRNA treatment, another IFI16 siRNA pool was applied to three additional donors (Supplementary Fig. 1b,c). Of note, in one of the donors we were unable to achieve sufficient knockdown with the siRNA targeting IFI16 and therefore observed no differences in interferon responses, further arguing against the possibility of unspecific effects of the siRNA treatment. Thus, IFI16 enhances the capacity of macrophages to sense HIV and to initiate an immunological response.

We hypothesized that lack of innate immune activation directed by IFI16 could lead to impaired viral control in macrophages. To test this, we knocked-down IFI16 in MDMs and investigated the levels of HIV production 6 days post-infection. We found that an average IFI16 reduction of 70% increased production of the macrophage-tropic HIV Bal strain by about two orders of magnitude (Fig. 1d). Similar findings were observed using another set of IFI16 specific siRNAs where a 3-fold enhancement was achieved (Supplementary Fig. 1d,e).

To better understand how IFI16 regulates the innate immune responses to viral infection, we utilized the CRISPR-Cas9 technology to generate knockout gene variants in human THP-1 cells, a monocytic cell line that adopts a macrophage-like phenotype on PMA differentiation. Single cell clones carrying IFI16 mutations were generated based on three different guide RNAs (Supplementary Fig. 2a,b). Verification of genetic KO was carried out by Western blot on PMA-treated THP1 cells (Supplementary Fig. 2c,d) and by single clone sequencing (Supplementary Fig. 2e). The THP-1 clones harbouring KO mutations in genes encoding STING and cGAS have been described earlier^37^.

Unlike HIV, members of the herpesviridae trigger robust innate immune responses in macrophages^6^,^7^,^9^. We have previously reported important roles for IFI16 in stimulating interferon responses to herpes virus infections using shRNA-directed knock-down^7^. We therefore sought to validate whether the KO cell lines exhibit a similar dependence on IFI16 for herpes virus-induced type I interferon production. In control PMA-treated THP1 cells, we observed elevated type I interferon secretion 18 h after infection with both herpes simplex virus 1 (HSV-1; Fig. 1e) and human cytomegalovirus (hCMV; Fig. 1f). This response was completely abolished in THP1 cells lacking cGAS and STING, and only minor residual induction of type I interferons in IFI16 KO cells was observed (Fig. 1e,f). Using a higher MOI of HSV1, we were able to explore interferon secretion at earlier time points (2–8 h). The results confirmed that IFI16 is required for potent early immune activation following HSV-1 infection (Fig. 1g).

To exclude potential off-target effects of the IFI16-directed CRISPR knockout, we evaluated innate immune responses to the paramyxovirus Newcastle disease virus (NDV), an RNA virus known to trigger RIG-I activation^38^,^39^. In this case, type I interferon and TNF-α responses were not affected by knocking out IFI16 (Supplementary Fig. 3a,b). Additionally, to exclude saturating effects of high NDV titres, we confirmed our results using sequential dilutions of viral inoculums (Supplementary Fig. 3c). Thus, IFI16 specifically enhanced the capacity of macrophages to sense infection by DNA-containing or −producing viruses.

### Early and robust activation of STING is regulated by IFI16

**Figure 2:**
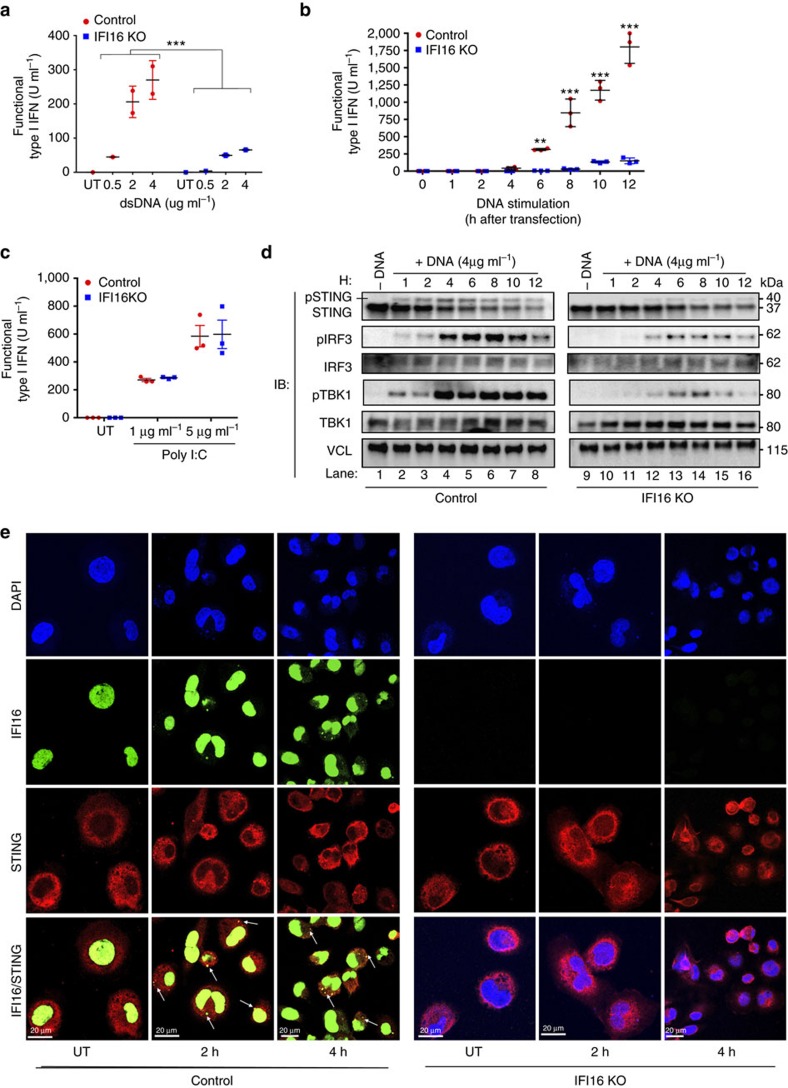
Cytosolic DNA sensing and efficient innate signalling is dependent on IFI16. (**a**) Control and IFI16 CRISPR KO THP-1 cells were transfected with dsDNA at various concentrations and interferon induction measured after 6 h. (**b**,**c**) Control and IFI16 KO cells were transfected with dsDNA (4 μg ml^−1^) at indicated time-points (**b**) or poly (I:C) (1 μg ml^−1^ or 5 μg ml^−1^) for 20 h (**c**), hereafter supernatants were evaluated for type I interferon expression. (**d**) Whole cell lysates from control or IFI16 KO cells stimulated with dsDNA (4 μg ml^−1^) at indicated time-points were subjected to immunoblotting using antibodies against STING, pIRF3, pTBK1, total TBK, total IRF3 and vinculin (VCL) as loading control. (**e**) Control or IFI16 KO cells were transfected with dsDNA (4 μg ml^−1^) for two and four hours. The cells were fixed and stained with anti-IFI16 (Green) and anti-STING (Red) specific antibodies. DNA was visualized with DAPI (blue). Data represent mean±s.d. of biological triplicates, representative of three independent experiments. Unpaired *t*-test corrected for multiple comparisons using Holm–Sidak was been performed to evaluate the significance. *P<0.05; **P<0.01; ***P<0.001.

A reliable approach to trigger immune activation by cGAS and STING is liposomal transfection of synthetic DNA of various structures. We therefore transfected control and IFI16 KO cells with different types and concentrations of synthetic DNA and evaluated early type I interferon secretion. Both HSV-1 60mer (dsDNA) and herring testis DNA (HT-DNA) induced robust interferon responses in control THP1 cells. In comparison, THP1 cells lacking IFI16 showed a significantly weaker interferon response (Fig. 2a; Supplementary Fig. 4a). Structured single-stranded HIV DNA, previously shown to trigger interferon signalling^5^, resulted in a similar interferon response as observed for HT-DNA and dsDNA (Supplementary Fig. 4b). Lipofectamine transfection alone did not induce a substantial amount of type I interferon (Supplementary Fig. 4c).

To further validate these observations, we transfected control and IFI16 KO THP1 cells with dsDNA and assessed type I interferon secretion over a 12 h time course. In control cells, we observed a continuous increase in interferon responses, whereas IFI16 KO cells depicted a significantly attenuated and delayed interferon response (Fig. 2b). This impairment in THP1 cells lacking IFI16 was even more pronounced when we evaluated expression of the IRF3-dependent target gene *CXCL10,* which was completely absent in IFI16 KO cells (Supplementary Fig. 4d). In contrast, transfection with polyI:C, that activates the RIG-I pathway, induced normal interferon (Fig. 2c) and TNF-α responses (Supplementary Fig. 4e) in both control and IFI16 KO cells. The minimal interferon response observed in IFI16 KO THP1 cells could be due to effects on other innate signalling pathways. However, when we pre-treated control and IFI16 KO cells with the TBK1/IKKɛ kinase inhibitor BX-795 before DNA transfection we observed a complete block of type I interferon responses (Supplementary Fig. 4f). To confirm that DNA transfection did not affect kinases other than TBK1 in the interferon induction pathway, we also evaluated interferon responses in TBK1 KO THP1 cells (Supplementary Fig. 4g). TBK1 KO cells did not produce type I interferon on transfection with dsDNA, in constrast to control THP1 cells (Supplementary Fig. 4h). To validate these findings in primary cells, we knocked-down IFI16 in MDMs using siRNA (Supplementary Fig. 5a) and transfected with dsDNA 48 h after the final siRNA treatment. Efficient IFI16 depletion was associated with significantly reduced interferon responses following transfection with dsDNA but not polyI:C (Supplementary Fig. 5b,c).

These observations were confirmed in additional THP1 IFI16 KO clones from a total of three different gRNA sequences (Supplementary Fig. 2b). All clones responded normally to polyI:C transfection or NDV infection but showed strongly impaired type I interferon responses to dsDNA (Supplementary Fig. 6a–e). Type I interferon responses were also absent upon dsDNA treatment in THP1 cells lacking cGAS or STING (Supplementary Fig. 6f). Finally, we reconstituted IFI16 expression by lentiviral gene delivery and demonstrated a partly restored IFI16 expression 48 h after transduction (Supplementary Fig. 6g). These cells gained the capacity to respond to dsDNA transfection observed by increased production of type I interferon, whereas the response to poly(I:C) transfection was unaltered (Supplementary Fig. 6h). Of note, we observed that lentiviral gene delivery of eGFP did not induce interferon production in IFI16 KO cells, however, it slightly increased interferon response following DNA stimulation in control cells (Supplementary Fig. 6h).

We speculated that the attenuated interferon responses observed in IFI16 KO THP1 cells could be due to impaired regulation of the STING signalling cascade. To evaluate this, we performed immunoblotting for STING, phospho-TBK1 and phospho-IRF3. We observed that transfection of cells with dsDNA resulted in the emergence of a slow migrating form of STING (Supplementary Fig. 7a, left side), possibly due to phosphorylation^40^. To confirm this we treated samples with alkaline phosphatase before SDS–PAGE electrophoresis. This resulted in disappearance of the slower migrating signal (Supplementary Fig. 7a, right panel). Next, control and IFI16 KO THP1 cells were transfected with dsDNA and evaluated for the appearance of the phosphorylated STING band. In control cells, this signal appeared after one hour and peaked between 4 and 6 h p.t. (Fig. 2d, upper left panel). In contrast, cells lacking IFI16 produced a very faint and transient signal after 4–6 h p.t. (Fig. 2d, upper right panel). Evaluation of TBK1 phosphorylation further indicated that control cells responded rapidly to dsDNA, whereas cells lacking IFI16 had a delayed and transient phosphorylation pattern (Fig. 2d). This attenuated response was also apparent IRF3 phosphorylation where control THP1 cells induced phospho-IRF3 after 1 h and peaked 8 h p.t., whereas IFI16 KO cells demonstrated at least a 4 h time delay and a weaker signal for phospho-IRF3 (Fig. 2d, lane 2–4 versus lane 10–12).

We then employed confocal microscopy to visualize the kinetic of STING activation as assessed by STING puncta formation after dsDNA transfection in both control and IFI16 KO cells. In control THP1 cells, IFI16 and STING colocalized in distinct spots with saturated DAPI staining, indicating transfected dsDNA, which was completely absent in cells lacking IFI16 (Fig. 2e). Furthermore, STING specks were clearly observed in control cells 2 h p.t., which further increased at 4 h (Fig. 2e). By contrast, THP1 cells lacking IFI16 formed no detectable specks at 2 h and very few at 4 h p.t. (Fig. 2e). This difference was significant when STING puncta were counted in multiple cells (Supplementary Fig. 7b,c). Altogether these data demonstrate that macrophages, which express cGAS and STING but lack IFI16, do not have the capacity to initiate early and robust STING signalsome activation in response to cytosolic dsDNA.

### STING dimerization and phosphorylation is controlled by IFI16

**Figure 3:**
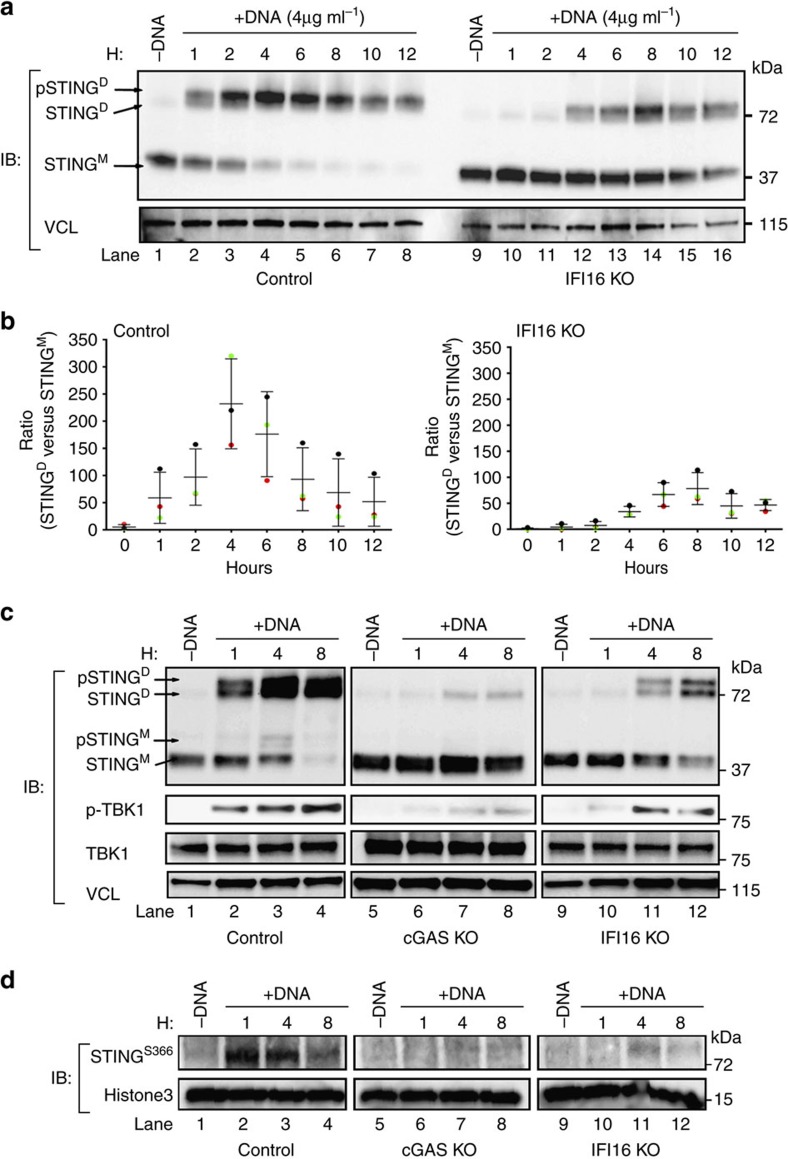
STING dimerization and phosphorylation is dependent on IFI16. (**a**,**b**) Control and IFI16 KO THP-1 cells were stimulated with dsDNA (4 μg ml^−1^) at indicated time-points and whole cell lysates were subjected to immunoblotting of STING dimerization by semi-native gel electrophoresis. Vinculin (VCL) was used as loading control. (**b**) The quantification of band intensity of STING^Dimer^ versus STING^Monomor^ was done using ImageJ software of three independent experimental setups. (**c**,**d**) Control, cGAS KO and IFI16 KO cells were stimulated with dsDNA (4 μg ml^−1^) at indicated time-points and whole cell lysates were subjected to both semi-native gel electrophoresis and standard SDS–page. Membranes were probed with antibodies against STING, p-TBK1 and VCL (**c**) or phospho-specific STING Ser^366^ and Histone3 as loading control (**d**). Data presented in (**a**,**c**) are representative of at least three independent experiments, whereas data in (**d**) is representative of two independent experiments.

Recently, it has been shown that dimerization of STING through cGAMP interaction precedes active TBK1 phosphorylation of STING and IRF3 (ref. 21). Thus, we hypothesized that IFI16 participates in regulating STING dimerization and subsequently TBK1/IRF3 activation. To test this, we utilized an in-house STING dimerization protocol^37^ that allows investigation of STING dimerization and phosphorylation after dsDNA transfection. To pinpoint STING dimerization, all experiments were conducted under semi-native conditions, as reducing conditions would disrupt the dimerization but not the phosphorylation signal of STING (Supplementary Fig. 7d). When control THP1 cells were stimulated with dsDNA, an immediate dimerization signal was observed that peaked in intensity after 4 h (Fig. 3a, lane 2–4). This signal was both delayed and less intense in THP1 cells lacking IFI16 (Fig. 3a, lane 10–14). On the basis of three individual experiments, we calculated that STING dimerization formation peaked at 4 h post dsDNA transfection in control THP1 cells, whereas cells lacking IFI16 demonstrated a delay of at least 4 h (Fig. 3b). These observations were confirmed in a IFI16 knockout clone generated with a different gRNA (Supplementary Fig. 7e).

We next sought to determine whether the delay in STING activation and phosphorylation were comparable in IFI16 and cGAS KO THP1 cells. As expected, control cells produced rapid STING dimerization after 1 h. The intensity of the dimerization signal further increased 4 h p.t. following a fading signal for STING monomer and increased signal for phospho-TBK1 (Fig. 3c, lane 2–4). As expected, cGAS KO THP1 cells generated a very faint STING dimerization signal (Fig. 3c, lane 6–8), as well as a weak signal of phosphorylated TBK1 (Fig. 3c, lane 7). In cells lacking IFI16, STING dimerization and phosphorylation was not observed until 4 h post-transfection, in which case the signal intensity was inferior to the signals observed for the control cells (Fig. 3c; lane 2–3 versus 10–11). In parallel to the delayed and reduced dimerization of STING, we also observed that THP1 cells lacking IFI16 did not induce TBK1 phosphorylation before 4 h p.t (Fig. 3c, lane 11).

**Figure 4:**
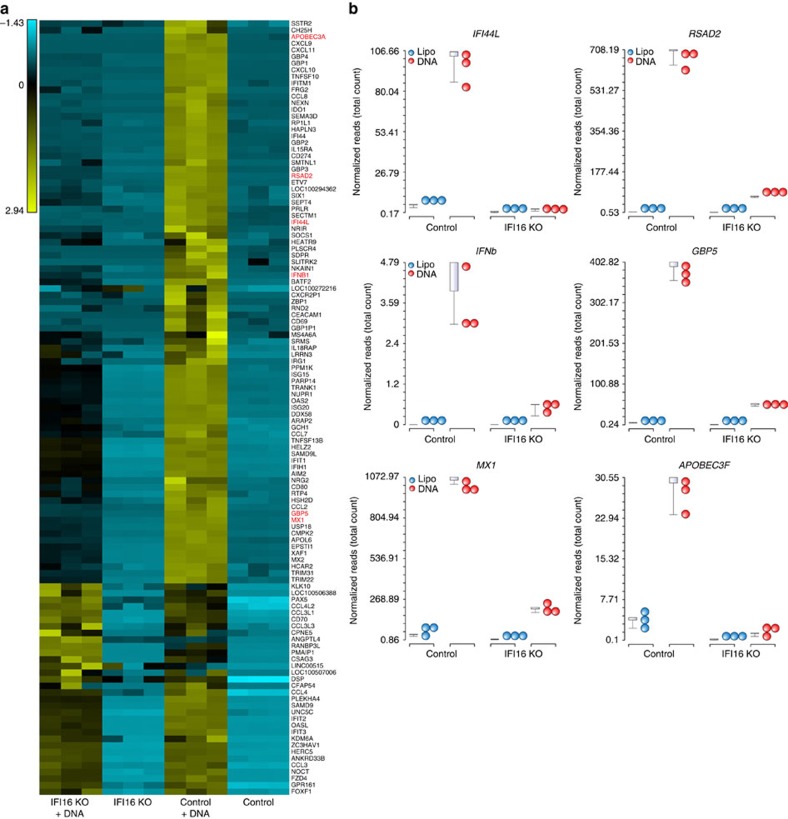
ISG expression profile in IFI16-deficient cells. (**a**) Control and IFI16 KO cells were stimulated with dsDNA (4 μg ml^−1^) for 6 h before extracting total RNA and analysing differentially expressed genes on ProtonIon with Partek Gene Specific Analysis Algoritm. (**b**) Total gene expression (number of reads normalized to total reads) are presented for the 6 red-marked genes: *IFI44L, VIPERIN, IFNB1, MX1, APOBEC3F* and *GBP5.* The box represents interquartile range, with the line in the middle representing the median while the whiskers symbolize 90–10% range.

Specific phosphorylation at STING Ser^366^ is essential for downstream signalling to IRF3 and its activation^21^,^40^,^41^. Thus, we evaluated STING phosphorylation using a phospho-specific antibody^21^ targeting Ser^366^. We confirmed that control cells stimulated with dsDNA induced a clear signal after 1 h, which remained elevated until 8 h p.t. (Fig. 3d, lane 2–4). This signal was absent in cGAS KO cells (Fig. 3d, lane 7) and strongly attenuated in IFI16 KO cells (Fig. 3d, lane 11). The attenuation and delay of STING activation observed in IFI16 KO cells would influence the capacity of macrophages to mount an early antiviral response, as indicated in Fig. 1. To complement this, we also evaluated RNAseq gene expression profiles in control and IFI16 KO THP1 cells stimulated with dsDNA for 6 h. We found that a total of 117 immune genes were induced at least 10-fold in control cells after stimulation; however, more than half of these genes were not upregulated in IFI16 KO cells (Fig. 4a). Closer examination of six classical type I interferon stimulatory genes (ISGs) showed a homogeneous increase of RNA reads in each biological sample of control macrophages stimulated with dsDNA, whereas IFI16 KO cells showed little if any differences (Fig. 4b). In conclusion, these data support that IFI16 controls STING activation at or upstream of STING phosphorylation.

### IFI16 recruits TBK1 to STING to initiate IRF3 activation

**Figure 5:**
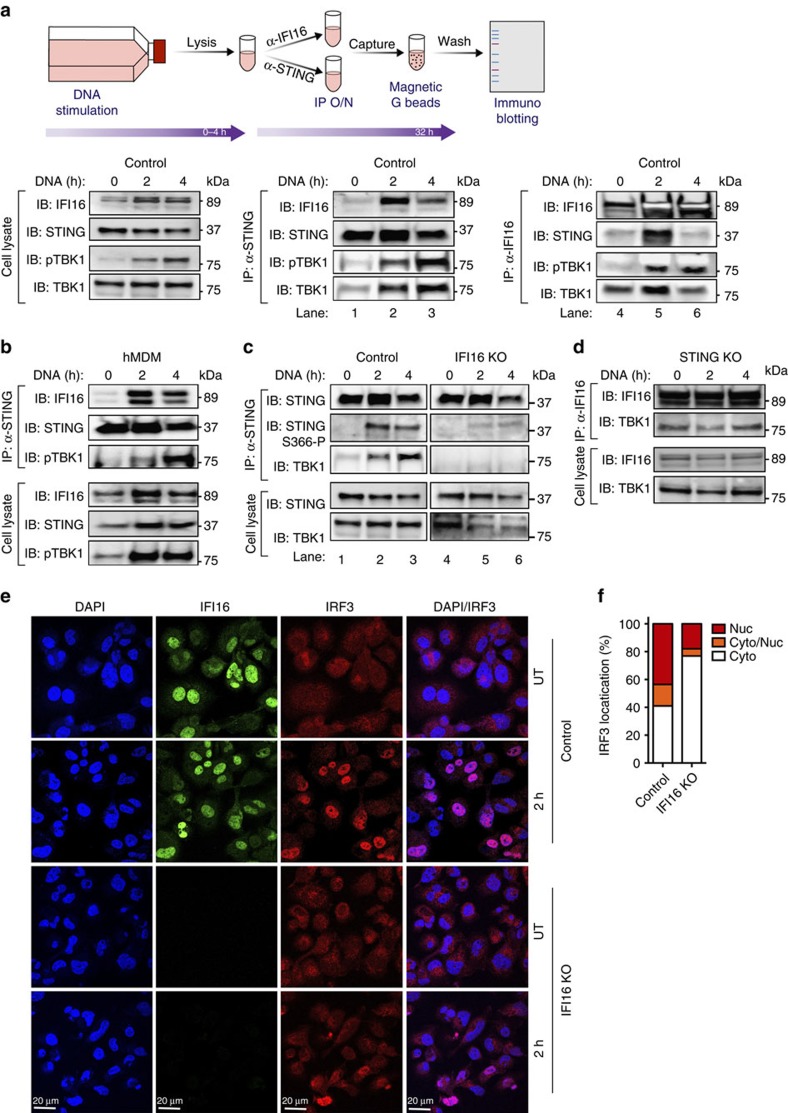
Recruitment of TBK1 to STING is dependent on IFI16 interactions. (**a**) Schematic illustration of the workflow of co-immunoprecipitation experiments. Cleared cell lysates (CCL) of THP-1 cells stimulated with dsDNA (4 μg ml^−1^) for 2 and 4 h were subjected to over-night co-immunoprecipitation with antibodies indicated in each panel. Lysates from control cells were co-IP with STING (lane 1–3) or IFI16 (lane 4–6). Input and elutes were analysed by gel electrophoresis followed by immunoblotting (IB) with the indicated antibodies. (**b**) STING co-IP samples from primary human MDMs after IB with the indicated antibodies. (**c**) STING co-IP samples from control (lane 1–3) and IFI16 KO (lane 4–6) THP-1 cells after IB with the indicated antibodies. (**d**) IFI16 co-IP samples from STING KO THP-1 cells after IB with the indicated antibodies. Each blot is representative of three independent experiments. (**e**) Control or IFI16 KO cells were stimulate with dsDNA (4 μg ml^−1^) for 2 h, fixed and stained for DAPI (blue), anti-IFI16 (green) or anti-IRF3 (red) and subjected to confocal imaging at × 63 oil lens. (**f**) Quantification of IRF3 localization of at least 50 individual cells treated as described in **e**.

On the basis of the results above, we next examined whether IFI16 directly interacts with factors involved in the STING signalling pathway following dsDNA stimulation. We did this by performing immunoprecipitation (IP) of STING or IFI16 on cytosolic extracts from THP1 cells and MDMs stimulated with dsDNA (Fig. 5). In STING-IP samples we observed a robust signal for IFI16 2 h p.t. that decreased at 4 h p.t. (Fig. 5a, lane 2–3), whereas we were unable to pull down IFI16 in STING KO cells (Supplementary Fig. 8). We also observed a strong association between STING and TBK1, as well as phospho-TBK1, after DNA stimulation (Fig. 5a, lane 2). Consistent with these results, IP with IFI16 antibodies on cytosolic extracts resulted in a robust signal for STING 2 h p.t., which was no longer apparent at 4 h (Fig. 5a, lane 5–6). Additionally, we observed that IFI16 strongly associated with TBK1 even in absence of stimulation (Fig. 5a, lane 4). This interaction was specific, since TBK1 was not precipitated by anti-IFI16 in THP1 IFI16 KO cells (Supplementary Fig. 8). On dsDNA transfection, the phosphorylated form of TBK1 was also precipitated together with IFI16 (Fig. 5a, lane 5–6). These results were recapitulated in cell lysates from primary MDMs using the STING-IP protocol (Fig. 5b).

These data implied that IFI16 potentially functions as a bridge between STING and TBK1. To test this hypothesis, we next conducted a STING IP of control and IFI16 KO THP1 cells stimulated with dsDNA. Remarkably, TBK1 recruitment to STING was absent in cells lacking IFI16 and these cells failed to mount phosphorylation of STING at Ser^366^ (Fig. 5c, lane 5–6). In contrast, control THP1 cells demonstrated both TBK1 recruitment to STING and strong phosphorylation of STING at position Ser^366^ (Fig. 5c, lane 2–3). Next, we performed IP of IFI16 in STING KO THP1 cells and recapitulated the constitutive association between TBK1 and IFI16 (Fig. 5d). Collectively, these data suggest that IFI16 is important for recruitment of TBK1 onto STING following dsDNA stimulation.

Impaired STING and TBK1 interactions should result in reduced IRF3 activation and translocation to the nucleus. Indeed, confocal microscopy confirmed IRF3 accumulation in the nucleus of control but not of IFI16 KO cells (Fig. 5e,f). Taken together, these results suggest that IFI16 constitutively interacts with TBK1 and is able to recruit TBK1 to STING following DNA stimulation, thus supporting phosphorylation of STING and subsequent IRF3 activation.

### Impaired cGAMP production in IFI16-deficient cells

**Figure 6:**
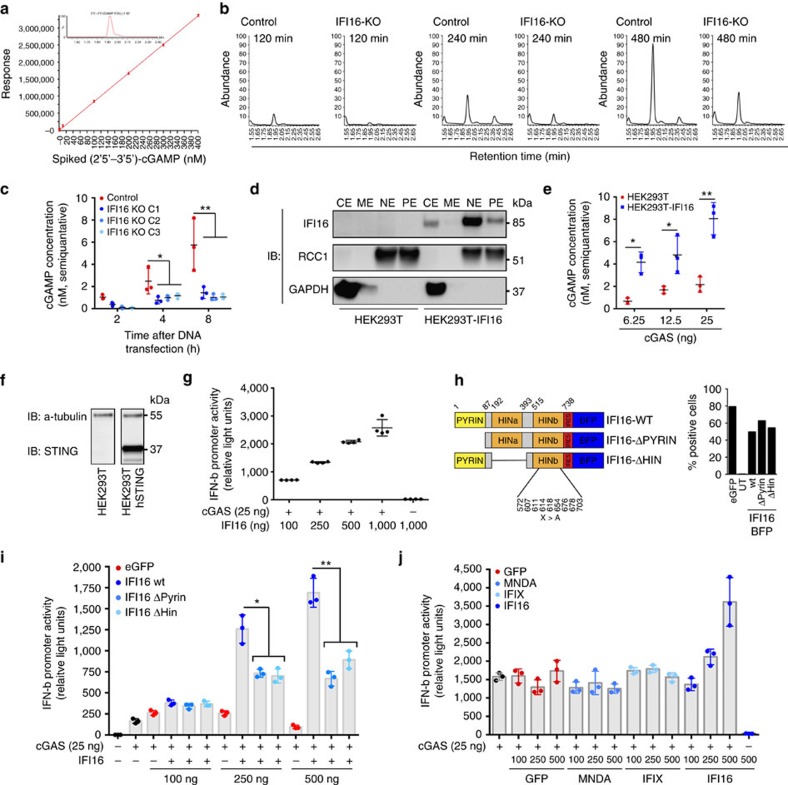
cGAMP production is regulated by IFI16. (**a**) External calibration curve of spiked (2′3′-3′5′)-cGAMP into cell extract before column purification were used to quantify cGAMP production in stimulated cells. The calibration curve was linear up to a concentration of at least 400 nM with an R2 of 0.991. The chromatogram demonstrates the peak detected using synthetic cGAMP. (**b**) LC-MS/MS chromatograms of whole cell lysates from control and IFI16 KO THP-1 cells stimulated with dsDNA for 2, 4 or 8 h. (**c**) Quantitative LC–MS/MS analysis of control and IFI16 KO THP-1 of three individual single clones. (**d**) Immunoblotting of HEK29T with or without stable transduction of human IFI16 (CE, cytoplasmic extract; ME, membrane extract; NE, nuclear extract; PE, pellet extract). (**e**) Quantitative LC–MS/MS analysis of HEK293T with or without stable transduction of human IFI16 24 h after transfection with increasing doses of cGAS expressing plasmid. (**f**) Immunoblotting of HEK293T with or without stable transduction of human STING. (**g**) HEK293T^STING^ cells were transfected with cGAS expressing plasmid (25 ng well^−1^) and increasing doses of IFI16 expressing plasmid (0, 250, 500, 750 and 1,000 ng well^−1^). STING activation was evaluated 24 h later by measuring expression of an IFN-β promoter Firefly gene normalized to a beta-actin promotor Renilla gene. (**h**) Diagram of IFI16 domains and the two different IFI16-mutants used to transient express IFI16 protein in HEK293T stable expressing human STING. An eGFP expressing plasmid was used as negative control. Transfection efficiencies were evaluated by measuring eGFP or BFP by Flow cytometry. (**i**) HEK293T^STING^ cells were transfected with cGAS expressing plasmid (25 ng well^−1^) and increasing doses of plasmids expressing wt, Pyrin or Hin IFI16 mutant. (**j**) HEK293T^STING^ cells were transfected with cGAS expressing plasmid (25 ng well^−1^) and increasing doses of plasmids expressing Pyrin containing proteins; MNDA, IFIX or IFI16. Data in (**c**,**e**,**g**,**i**,**j**) represent mean±s.d. of biological triplicates from three independent experimental setups. Unpaired *t*-test corrected for multiple comparisons using Holm–Sidak was performed to evaluate the significance. For data in (**i**) one-way ANOVA was performed to evaluate significance. *P<0.05; **P<0.01, ***P<0.001.

A prerequisite for DNA-stimulated STING activation and TBK1 recruitment is activation of cGAS to produce cGAMP (ref. 16). In dendritic cells, this process has been suggested controlled by a cellular cofactor PQBP1 during sensing of retroviral DNA (ref. 33). Thus, we hypothesized that the lack of interferon induction observed in IFI16 KO THP1 cells was because of the absence of IFI16 supporting cGAS activity following DNA binding. To evaluate this, we measured mammalian 2′5′-3′5-cGAMP (hereafter cGAMP) by LC-MS/MS analysis (Fig. 6a). Interestingly, IFI16 KO THP1 cells had lower cGAMP production than control cells following DNA transfection (Fig. 6b). Using the external calibration curve of synthetic cGAMP (Fig. 6a) we were able to quantify cGAMP production over multiple experiments, confirming that control THP1 cells produced significantly more cGAMP after DNA challenge than IFI16 KO clones (Fig. 6c).

These data indicated that IFI16 directly supports the capacity of cGAS to sense DNA and activates the signalling complex. To confirm this in another system, we used HEK293T cells, which do not express IFI16, cGAS or STING, but do activate the IFN-β promoter in responsive to plasmid DNA transfection upon overexpression of STING and cGAS (ref. 42). Using Sleeping Beauty DNA transposon technology, we generated a HEK293T cell model stably expressing human IFI16. These cells demonstrated a subcellular distribution of IFI16 reminiscent of macrophages (Fig. 6d), with the majority of IFI16 accumulating in the nucleus, but a significant portion in the cytoplasm (Fig. 6d, CE and NE lanes). We titrated cGAS into these cells to examine whether IFI16 supported cGAMP production by cGAS in response to sensing of the transfected plasmids. Indeed, HEK293T^IFI16^ cells generated significantly more cGAMP measured by LC-MS/MS than normal HEK293T cells (Fig. 6e).

We examined next whether overexpression of IFI16 in combination with cGAS had any synergistic effect in the HEK293T stable expressing human STING (Fig. 6f). Overexpression of IFI16 alone did not render cells responsive to plasmid DNA, whereas co-expression of cGAS alone resulted in robust IFN-β promoter-stimulated luciferase activity (Fig. 6g). When we titrated increasing doses of IFI16 in HEK293T^STING^ expressing cGAS we observed prominent dose-response effects of IFI16, indicating that IFI16 enhances cGAS activation (Fig. 6g).

To identify the domain responsible for triggering cGAS activation, we transiently transfected HEK293T^STING^ cells with two different IFI16 mutants: a PYRIN-domain mutant and a DNA-binding mutant, in which we deleted the HIN-a domain and made specific HIN-b site-directed mutagenesis^31^ in residues essential for DNA binding (Fig. 6h). All IFI16 plasmids contained an IRES-BFP cassette to control for gene expression by flow cytometry (Fig. 6h). When we overexpressed any of the IFI16 constructs in a HEK293T^STING^ background no IFN-β promoter activity was detected. However, expression of cGAS in combination with wildtype IFI16 significantly elevated IFN-β promoter activity above control plasmid expression (Fig. 6i). Additionally, when each of the IFI16 mutants were co-expressed together with cGAS, we observed significantly reduced IFN-β promotor activity compared with IFI16 wildtype. To examine whether this was a specific function by the IFI16 PYRIN domain, we overexpressed two other PYRIN-domain containing proteins also reported as sensors of DNA (MNDA and IFIX)^43^ in HEK293T^STING^ cells. However, increasing doses of MNDA and IFIX did not increase IFN-β promoter activity (Fig. 6j). To further confirm specificity of IFI16 for the STING pathway, we activated the IFN-β promoter by overexpression of the phospho-mimic IRF3 mutant IRF3-5D, or the adaptor protein MAVS (Supplementary Fig. 9). Co-expression of IFI16 did not elevate IFN-β promotor activity supporting that the function of IFI16 is specific to the STING pathway. In conclusion, IFI16 augments cGAS-dependent responsiveness to DNA, and this is dependent on the PYRIN and HIN domains of IFI16.

### IFI16-deficient cells are unaffected by cGAMP stimulation

**Figure 7:**
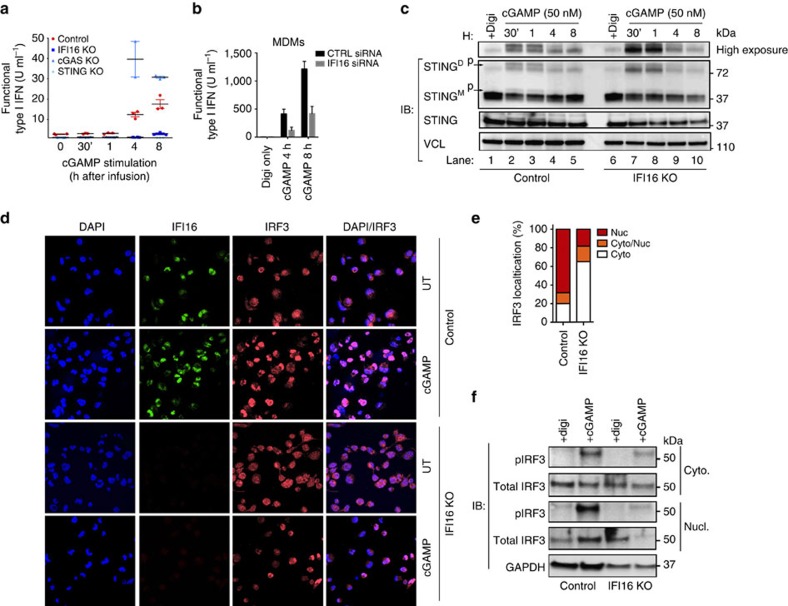
IFI16 regulates cGAMP-mediated STING activation. (**a**) Control, IFI16, cGAS, STING KO THP-1 cells or (**b**) MDMs with IFI16 siRNA knockdown, were infused with cGAMP (50 nM) at indicated time-points and subsequently evaluated for type I interferon secretion. (**c**) STING dimerization analysis by semi-native western blotting. Upper lane represents an overexposure of the dimer STING band. Total STING was run on a separate SDS–Page gel. (**d**) Control and IFI16 KO cells were infused with cGAMP (50 nM) for 2 h, fixed and stained for DAPI (blue), IFI16 (green) and IRF3 (red). (**e**) IRF3 translocation from cytoplasm to nuclear saturation were quantified by counting >50 separate images of control or IFI16 KO cells 2 h post cGAMP infusion. (**f**) Subcellular fractions of control and IFI16 KO cells stimulated with 50 nM cGAMP for 1 h were immunoblotted for phosphorylated IRF3 and total IRF3 in cytosolic (cyto) and nuclear (nucl) fractions. Data in (**a**,**b**) represent mean±s.d. of biological triplicates from (**a**) three independent experimental setups or (**b**) one donor; (**c**–**f**) data is representative of one of three independent experiments.

Based on the results above, we hypothesized that limited cGAMP production might not be the only factor contributing to impaired STING activation. We therefore sought to determine whether the immune response in IFI16 KO THP1 cells was normalized when bypassing the cGAS-DNA sensing mechanism. We explored this by stimulating cells directly with cGAMP. As expected, control cells and cGAS KO THP1 cells demonstrated a clear type I interferon response 4 and 8 h after infusion with cGAMP, whereas STING KO cells were insensitive to cGAMP stimulation (Fig. 7a). Interestingly, IFI16 KO cells behaved in a similar manner as STING KO cells (Fig. 7a). In MDMs, cGAMP infusion resulted in strong type I interferon responses in cells treated with scrambled siRNA whereas the response was significantly lower in MDMs treated with IFI16-specific siRNA (Fig. 7b). Using confocal microscopy we observed that control cells stimulated with cGAMP generated specific STING patterns, including multiple small cytoplasmic puncta and larger aggregations, in ER-like formations (Supplementary Fig. 10). In contrast, IFI16 KO cells generated small aggregates in ER but no cytoplasmic spots (Supplementary Fig. 10), indicating that IFI16 participates in regulating the function of STING downstream of cGAMP activation.

Next, we evaluated the kinetics of STING dimerization and phosphorylation in cells stimulated with cGAMP infusion. Digitonin alone did not result in STING dimerization (Fig. 7c). However, on stimulation with cGAMP, control THP1 cells demonstrated effective shift in STING dimerization after just 30 min (Fig. 7c). Unexpectedly, IFI16 KO THP1 cells produced a strong STING dimerization signal. However, this was not phosphorylated as observed for the control cells (Fig. 7c, high-exposure plot; lane 2–3 versus 7–8), suggesting a lack of TBK1 recruitment to the STING dimerization complex in absence of IFI16, which support our earlier findings (Fig. 5). This reduced STING phosphorylation would propose impaired IRF3 activation. This was confirmed by confocal microscopy visualizing IRF3 nuclear translocation one hour after cGAMP infusion (Fig. 7d). When multiple cells were evaluated, we found notably increased nuclear accumulation of IRF3 in control versus IFI16 KO cells (Fig. 7e). These results are supported by immunoblotting for phospho-IFR3 in lysates from control and IFI16 KO cells stimulated with cGAMP demonstrating strong signals for IRF3 phosphorylation in cytoplasmic fractions of control cells and a very faint signal in IFI16 KO cells (Fig. 7f). In nuclear fractions we observed an about 50% reduction of the signal for phospho-IRF3 in IFI16 KO cells (Fig. 7f), recapitulating the observations from the confocal microscopy.

Altogether, these results indicate that IFI16 not only cooperates with cGAS to promote cGAMP production but also has a key function downstream of cGAMP-STING interaction involving its recruitment of TBK1 to STING for phosphorylation.

### PYRIN domain is essential for cGAMP-mediated STING signalling

The data presented suggested that IFI16 and TBK1 cooperate for effective activation of STING. To determine at which step IFI16 acts in the cGAMP-activated pathway, we measured STING dimerization and phosphorylation in control, IFI16 KO, and TBK1 KO THP1 cells. First, we confirmed that TBK1 KO cells stimulated with cGAMP did not produce type I interferon (Supplementary Fig. 11a). Multiple THP1 IFI16 KO clones were also evaluated for their response to low (50 nM) and high (400 nM) cGAMP infusion, demonstrating minimal type I interferon production (Supplementary Fig. 11b,c). Furthermore, reconstitution with IFI16 by lentiviral transduction before cGAMP infusion, demonstrated a partly restored interferon respond to cGAMP, which was not observed using a lentiviral transduction with eGFP (Supplementary Fig. 11d,e). The degree of interferon induction in the reconstituted cells did; however, not reach similar levels as control cells, probably due to a lower overall expression of IFI16 in the reconstituted KO cells (Supplementary Fig. 11d).

It is known that STING can also be activated by bacterial cyclic-di-nucleotides such as cyclic-di-AMP (refs 10, 44, 45). We found that THP1 cells infused with high and low doses of cyclic-di-AMP responded to high doses of c-di-AMP in an IFI16 dependent manner (Supplementary Fig. 11f).

**Figure 8:**
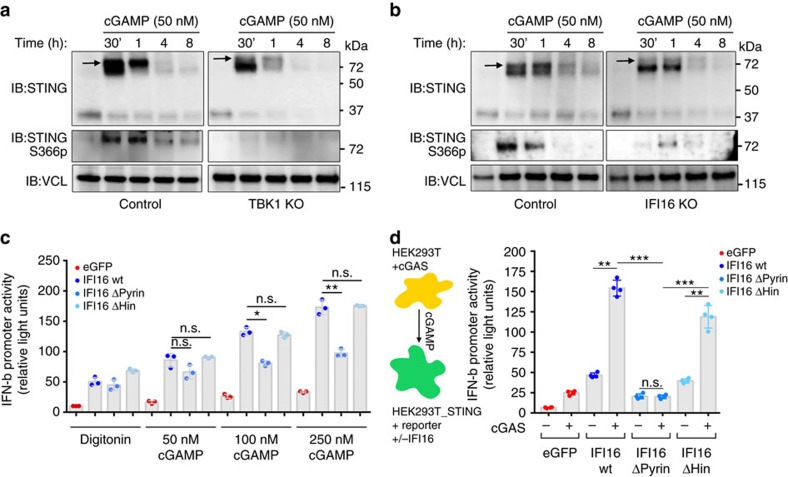
IFI16 regulates STING activation through its PYRIN domain. (**a**) Control and TBK1 KO or (**b**) Control and IFI16 KO THP-1 cells were infused with cGAMP (50 nM) for 30 min, 1, 4 and 8 h and whole cell lysates was used to evaluate STING dimerization (upper panel) and specific STING phosphorylation at Ser^366^ (lower panel). Black arrows represent the phosphorylated form of the STING dimer. (**c**) HEK293T^STING^-IFI16 expressing cells were infused with cGAMP (range from 50 to 250 nM) for 16 h and the degree of STING activation was evaluated by measuring expression of an IFN-β promoter Firefly gene normalized to a beta-actin promotor Renilla gene. (**d**) HEK293T- cGAS expressing cells were co-cultured with HEK293T^STING^ that had been transfected with eGFP or one of the three IFI16 variants. Twenty-four hours after culturing cGAMP transfer and STING activation was evaluated by measuring expression of IFN-β promoter Firefly gene normalized to beta-actin promotor Renilla gene. Data represent mean±s.d. of biological triplicates, representative of three independent experiments. Unpaired *t*-test corrected for multiple comparisons using Holm–Sidak was performed to evaluate the significance. *P<0.05; **P<0.01.

We next investigated STING dimerization and observed that TBK1 KO cells produced STING-dimers at levels similar to control cells at early time points p.i. (Fig. 8a), as well as to IFI16 KO cells (Fig. 8b). However, in both cases we did not detect the slower migrating phosphorylated band seen in the control cells. We further, evaluated the degree of direct STING phosphorylation at Ser^366^ and observed control THP1 cells mounting a robust phospho-signal 30 min to 1 h after cGAMP infusion (Fig. 8a,b) but as expected, no signal was detected in TBK1 KO cells (Fig. 8a). Moreover, in IFI16 KO cells we merely observed a very weak STING Ser^366^ phospho-signal 1 h after stimulation (Fig. 8b).

The IFI16 PYRIN domain engages in protein–protein interactions, while the HINb domain is central for DNA binding^3^,^31^. To identify the domain(s) responsible for triggering STING activation after cGAMP stimulation, we next evaluated IFN-β promoter activity in HEK293T^STING^ cells overexpressing the various IFI16 constructs (Fig. 6i). In cells expressing wildtype IFI16, we observed a significant increase in the IFN-β promoter-stimulated luciferase activity response to cGAMP compared with cells expressing eGFP only, which was saturated between 500 and 1,000 nM cGAMP (Supplementary Fig. 12). Interestingly, the ΔHin-IFI16 mutant augmented reporter gene expression to the same extent as wildtype IFI16, whereas the ΔPyrin-IFI16 mutant was impaired in enhancing cGAMP-stimulated STING-dependent IFN-β promoter activation (Fig. 8c). To exclude the possibility that we oversaturated cell activation levels by infusing high doses of recombinant cGAMP, we also investigated the response of cGAMP production in HEK293T cells and the possible transfer to other cells through gap-junctions^42^. We transfected HEK293T cells with cGAS-expressing plasmid and subsequently co-cultured them with HEK293T^STING^ co-expressing one of the three IFI16 variants. Co-culturing cGAS-expressing cells with HEK293T^STING^ resulted in minor IFN-β promoter-stimulated luciferase activity (Fig. 8d). Co-expressing eGFP from control plasmid resulted in low IFN-β activity possible due to a direct STING activation^46^, but when these cells were co-cultured with cGAS-expressing cells the IFN-β promoter-stimulated luciferase activity significantly increased above background levels (Fig. 8d). When HEK293T^STING^-IFI16 cells were co-cultured with cGAS-expressing cells, the IFN-β promoter-stimulated luciferase activity significantly increased, once again indicating that IFI16 expression supports cGAMP-transferred activation of STING. A similar response was observed when we investigated the ΔHin-IFI16 mutant (Fig. 8d). Finally, overexpression of the ΔPyrin-IFI16 did not increase IFN-β promoter-stimulated luciferase activity (Fig. 8d), indicating that the protein–protein interaction domain of IFI16 is necessary for efficient activation and signalling of STING following cGAMP production.

## Discussion

DNA potently stimulates innate immune responses through a pathway dependent on the DNA sensor cGAS and signalling via STING, TBK1 and IRF3 to induce type I interferon expression^1^. Previously, several other DNA sensors have been proposed^2^. Here, we demonstrate a central role for IFI16 in innate DNA sensing in macrophages, and unveil a ‘two-step' interconnected process where IFI16 enhances both cGAS-mediated cGAMP production, and cGAMP-mediated activation of the STING signalsome (Supplementary Fig. 13).

The function of IFI16 as a DNA sensor was demonstrated more than five years ago^6^, and work by us and others suggested important roles for IFI16 in pathogen recognition^5^,^9^,^10^. However, after the discovery of cGAS as a DNA sensor and demonstration of a clear role of this protein in both men and mice^13^,^16^,^47^ less attention has been given to other proposed DNA sensors, including potential interactions with the cGAS-STING pathway. However, a recent report highlights that cGAS may need cofactors to achieve optimal binding to cytosolic DNA^33^. Contrary, a recent report observes unaltered DNA sensing in mice depleted for the locus containing PYHIN-encoding genes^48^. These results argue against a role for murine PYHIN proteins in the cGAS-STING pathway. However, since mice do not have a clear ortholog of IFI16, it is difficult to extrapolate the murine data to human cells.

Our present results reiterate the necessity of cGAS and IFI16 to drive interferon responses in the human macrophage-like cell line THP-1 during infection with HIV, HSV-1 and CMV or by stimulation with synthetic DNA. More interestingly, we demonstrated that IFI16 is also necessary for initiating an early and robust interferon response to cytosolic DNA, HIV and herpes infections, and cGAMP stimulation in primary human macrophages. Our results suggest that IFI16 and cGAS cooperate in macrophages to enable optimal production of cGAMP that allows STING dimerization and downstream signalling events. We speculate that IFI16 has a function as co-factor for cGAS, through its capacity to bind double-stranded DNA (Supplementary Fig. 13). This model is similar to what has been suggested for PQBP1 in human dendritic cells^33^. However, PQBP1 appears to be more specific as a co-factor for retroviral infections than IFI16, although the precise PAMP has not been identified.

We have previously demonstrated that monocytic undifferentiated THP-1 cells have very low basal expression of IFI16 but show high expression levels of cGAS. These cells do respond immunologically to cytosolic DNA (ref. 10). However, when differentiated into macrophage-like cells over a two-day period they drastically change morphology, and also display strong elevation of IFI16 expression and decreased cGAS expression, which correlates with elevated interferon and cytokine responsiveness compared to undifferentiated cells^10^. Nonetheless, DNA-driven IFN-β expression is independent of IFI16 in undifferentiated THP-1 cells^10^. This suggests that the core cGAS-STING pathway may use a panel of co-factors, including IFI16 and PQBP1, depending on the cellular context. Notably, the physiological relevance of the IFI16-dependent phenotype observed in PMA differentiated THP-1 cells is supported by the demonstration of a role for IFI16 in primary human monocyte-derived macrophages for both DNA stimulation and cGAMP infusion. It is also interesting to notice that in the study by Almine *et al*.^49^ an analogous function of IFI16 in human keratinocytes was observed. Primary keratinocytes treated with siRNA and HaCaT cells with IFI16-deficience lacked the capacity to produce interferon in respond to exogenous DNA and cGAMP. In comparison to our study, Almine *et al*. did however not observe that IFI16-deficiency affected production of cGAMP. These variances indicate cell-type specific differences for the fine-tuning of IFI16 function in respond to DNA.

The experimental design used here aimed to address a potential role for IFI16 in the cytosolic compartment. It is known that IFI16 is a multifunctional protein involved in nuclear as well as cytosolic processes, including sensing of foreign DNA in the nucleus^3^,^50^. Others have reported a role for IFI16 in transcriptional regulation of the IFN-β promoter^51^. Our results based on measurement of cGAMP production, STING dimerization and phosphorylation clearly demonstrate that human macrophages deficient of IFI16 have impaired capacity to initiate these processes and hence to stimulate IRF3 activation. Thus, in the PMA-THP-1 macrophage model, IFI16 has a function as co-factor for cGAS, most likely through its ability to bind DNA. Of note, based on other recent publications^9^,^11^ we cannot exclude that IFI16 may also act by stabilizing cGAMP and inhibiting its turnover. Further studies are warranted to address this possibility. In addition, when macrophages were stimulated with cGAMP directly, we identified a hitherto unknown function of IFI16, independent of its cooperative function for cGAS activity. Specifically, IFI16 was not necessary for STING to initiate dimer formation, but was important for recruitment of TBK1 to STING and phosphorylation of STING. A recent study shows that STING can be phosphorylated at numerous positions, where phosphorylation at Ser^366^ is important for downstream signalling to IRF3 (ref. 21). In addition, it has been proposed that activation of STING is associated with its trafficking from the ER to the ER-Golgi intermediate compartment^19^. In light of these data, it is interesting to note that even though STING dimerization occurred in IFI16-deficient cells with a delayed response, we observed less STING degradation in combination with reduced TBK1-directed phosphorylation. This could indicate that STING dimerization occurred in the ER, but did not traffic to the ER-Golgi intermediate compartment, where it is assumed to associate with TBK1 and leading to subsequent degradation of STING by the autophagy pathway^19^. However, further studies are warranted to determine whether STING translocation from the ER occurs in the absence of IFI16.

On the basis of our immunoprecipitation results, we propose that IFI16 constitutively binds TBK1. Upon cGAMP-triggered STING dimerization, IFI16 may then form a signalling complex with STING and TBK1 that allows TBK1 to phosphorylate STING. Furthermore, based on the results obtained using IFI16 mutants we propose that IFI16 promotes activation of the cGAMP-activated STING-TBK1 pathway by a process relying on PYRIN domain-dependent protein-protein interactions.

In conclusion, our data reveal that IFI16 is important for the DNA-activated cGAS-cGAMP-STING-TBK1 pathway in human macrophages, and that it acts at two levels in the upstream part of the pathway. IFI16 (i) augments cGAS-mediated production of cGAMP and (ii) enables cGAMP-mediated recruitment of TBK1 to STING. Our work thus identifies IFI16 as an important cofactor for cGAS in the initial steps of DNA sensing upstream of cGAMP production, and provides evidence for a novel appliance of a known PRR needed for downstream events in the STING signalling pathway. Finally, the results reveal that the activity of the cGAS-cGAMP-STING-TBK1 pathway can be fine-tuned by the cell-type and context-specific expression of the cellular factor IFI16.

## Methods

### Reagents

Cyclic [G(2′,5′)pA(3′,5′)p] (cGAMP) was obtained from BioLog. The dsDNA (HSV-1 60mer) was synthesize and annealed by DNA technology; sense strand 5′- CCA TCA GA AAG AG GTT TAA TA TTT TTG TGA GAC CAT CGA AGA GAG AAA GAG ATA AAA CTT; antisense 5′- AAG TTT TAT CTC TTT CTC TCT TCG ATG GTC TCA CAA AAA TAT TAA ACC TCT TTC TGA TGG. For more information see^6^. The ssDNA1 sequences was 5′-GTC TCT CTG GTT AGA CCA GAT CTG AGC CTG GGA GCT CTC TGG CTA ACT AGG GAA CCC ACT GCT TAA GCC TCA ATA AAG CTT GCC TTG AGT GCT TCA AGT AGT G-3′. For more information see^5^. Herring testis DNA was from Sigma Aldrich (D6898); BX795 (tlrl-bx7) and poly I:C (tlrl-pic) were both acquired from InvivoGen.

### Plasmids

IFI16 mutant plasmid constructs were originated from a pCDNA3 human IFI16-HA tagged expression construct kindly provided by Professor Andrew Bowie, Trinity College Dublin. Overlap extension PCR was used to construct a ΔPyrin domain (consisting of amino acids 87–729 of IFI16) or a ΔHIN-A domain+HIN-B domain specific mutations (consisting of amino acids 1–191 and 460–729 of IFI16 and the point mutations K572A, K607A, R611A, S614A, K618A, N654A, K676A, K678A, K703A). Each PCR product was then recloned into a BamHI –and XhoI-digested pCCL-PGK-eGFP (ref. 52) together with a PCR-amplified IRES-BFP fragment by NEBuilder HiFi DNA Assembly according to manufactures instructions. For illustration see Fig. 6h.

The mBanana-cGAS fusion construct was engineered by PCR amplification of mBanana and cGAS and subsequent cloning into a NotI-digested pT2/CMV-eGFP.SV40-neo^53^ by NEBuilder HiFi DNA assembly according to manufactures instructions.

### Cell culture

Human acute monocytic leukaemia cell line (THP-1) was cultured in RPMI 1640 (Lonza) supplemented with 10% heat inactivated fetal calf serum, 200 IU ml^−1^ Penicillin, 100 μg ml^−1^ Streptomycin and 600 μg ml^−1^ glutamine (hereafter termed RPMI complete). Mycoplasma infection was tested and ruled on a monthly basis using Lonza MycoAlert kit (LT07–703). To differentiate THP-1 cells into adherent phenotypically macrophages, cells were stimulated with 100 nM Phorbol 12-myristate 13-acetate (PMA, Sigma Aldrich 79346 5MG) in RPMI complete for 24 h before medium was refreshed with normal RPMI complete and allowed to further differentiate an additional day (hereafter defined as macrophages). Of note, the haplotype of STING in the THP-1 parental cell type has been identified to be HAQ (ref. 17).

Peripheral Blood Mononuclear cells (PBMCs) were isolated from healthy donors (blood donors gave written consent as accordingly to the ethical guidelines at Aarhus University Hospital) by Ficoll Paque gradient centrifugation (GE Healthcare). Monocytes were separated from PBMCs by adherence to plastic in RPMI 1640 supplemented with 10% AB-positive human serum. Differentiation of monocytes to macrophages was achieved by culturing in Dulbecco's Modified Eagle Medium (DMEM) supplemented with 10% heat inactivated AB-positive human serum; 200 I IU ml^−1^ Penicillin; 100 μg ml^−1^ Streptomycin and 600 μg ml^−1^ glutamine for 10 days in the presence of 10 ng ml^−1^ M-CSF (R&D Systems).

HEK293T cells were cultured in Dulbecco's Modified Eagle Medium (DMEM) supplemented with 10% heat inactivated FCS; 200 I IU ml^−1^ Penicillin; 100 μg ml^−1^ Streptomycin and 600 μg ml^−1^ glutamine. HEK-Blue IFN-α/β (InvivoGen) cells were cultured in DMEM supplemented with 10% heat inactivated FCS; 200 IU ml^−1^ Penicillin, 100 μg ml^−1^ Streptomycin, 600 μg ml^−1^ glutamine, 100 μg ml^−1^ normocin (InvivoGen), 30 μg ml^−1^ blasticidin (InvivoGen) and 100 μg ml^−1^ zeocin (InvivoGen).

TZM- bl indicator cells (kindly provided by Drs Kappes and Wu and Tranzyme Inc. through the NIH AIDS Reagent Program) were cultured in Dulbecco's Modified Eagle Medium (DMEM) supplemented with 10% heat inactivated FCS; 200 I IU ml^−1^ Penicillin; 100 μg ml^−1^ Streptomycin and 600 μg ml^−1^ glutamine.

### Viruses

Newcastle Disease Virus (NDV) was kindly provided by professor Peter Palese (The mount Sinai Hospital, USA); HSV1+17 and hCMV (AD169) viral strains were propagated in-house.

Replication competent HIV macrophage tropic strain Bal was generated in-house.

VSVg-pseudotyped Vpx-packed particles were generated by transfecting HEK293T cells with the plasmid pMD.2G and SIV3+ (containing all SIVmac proteins except Env, kindly provided by professor Gregory Towers, UCL). Supernatants were harvested after 48 h, concentrated through a 20% sucrose cushion. Viral pellets were resuspended in PBS, DNase treated and stored at −80 °C. Each Vpx prep was concentration determined by p24 ELISA and used at 125 pg p24 setup.

### Transduction of THP-1 cells with lentiviral CRISPR/Cas9

We employed the CRISPR/Cas9 system to generate a set of THP-1 single clones with specific gene knockouts. Specifically, we used a lentiviral CRISPR/Cas9 vector^54^ that encodes a codon-optimized nuclear-localized Cas9 gene N-terminally fused to the puromycin resistance gene via a T2A ribosome-skipping sequence. Additionally, the vector contains a human U6 promoter driving expression of a guideRNA (gRNA) consisting of a gene-specific CRISPR RNA (crRNA) fused to the *trans*-activating crRNA (tracrRNA) and a terminator sequence.

The gene-specific crRNA sequences cloned were: For Control KO cells we used the gene of beta-2-microglobulin (5′-GAGTAGCGCGAGCACAGCTA-3′); for IFI16 KO (#1; 5′-GTACCAACGCTTGAAGACC-3′), (#2; 5′-GTTCCGAGGTGATGCTGGTT-3′) or (#3; 5′- GACCAGCCCTATCAAGAAAG-3′); for cGAS KO (5′-GACTCGGTGGGATCCATCG-3′); for STING KO (5′-GAGCACACTCTCCGGTACC-3′); and for TBK1 KO (5′-GTCAGATTCTGGTAGTCCAT-3′).

VSVg-pseudotyped lenti-CRISPR virions were produced by transfecting HEK293T cells with the following plasmids: CRISPR/Cas9 vector, pMD.2G, pRSV-REV, and pMDlg/p-RRE. Viral supernatants were harvested after 72 h and used to transduce THP-1 cells by infection in the presence of 4 μg ml^−1^ polybrene. Transduced cells were selected with 2 μg ml^−1^ puromycin at 2 days post transduction. After two weeks a single cell suspension culture was established using limiting dilution. After three weeks individual clones were subjected to western blotting to confirm absence of the targeted gene products (see also Supplementary Fig. 1a). At least 15 clones were then assessed for proper cell proliferation and expansion, and dismissed if they were slow growing or increased cell death.

### Reconstitution of IFI16 by lentiviral transduction

For the IFI16 reconstitution, the lentiviral vector pCCL/PGK-IFI16 was generated by inserting a PCR amplified IFI16 fragment from pCCL/CMV-DH287-IRES-BFP into BamHI –and XhoI-digested pCCL-PGK-eGFP^49^ by NEBuilder HiFi DNA assembly.

Packaging plasmids pMD2.G, pRSV-Rev and pMDIg/pRRE were calcium phosphate-transfected together with pCCL/PGK-IFI16 into HEK293T cells. Vector-containing supernatants were harvested by filtration through a 0.45 μm filter (Sarstedt) and ultracentrifuged at RPM 25,000 at 4 °C for 2 h on a 20% sucrose cushion. Pellets were re-suspended in PBS and stored at −80 °C. Two hundred and fifty ng p24 of LV-IFI16 or LV-eGFP (control) inoculums were then used to infect PMA-undifferentiated THP1 IFI16 KO cells using 6 μg ml^−1^ polybrene. After 6 h, PMA was added and cells were allowed to differentiate into macrophages. After 48 h, cells were stimulated with DNA or cGAMP. Supernatant was harvest after 8 or 20 h for type I interferon bioassay and cells were lysed for verification of IFI16 expression by immunoblotting.

### Transfection of HEK293T cells

Human Embryonic Kidney 293T (HEK293T) cells were stably transfected with wild type human STING using the sleeping beauty-mediated transposition system. Human STING and SB100X encoding vectors were mixed with Polyethyleneimine in a 1:3 relationship and administered to the cells, which were allowed to incubate for 48-h. Cells were subsequently selected with 1 μg ml^−1^ puromycin for two weeks and allowed to expand before analysing stable expression of STING via western blotting.

### DNA/RNA stimulation of cells

Standard stimulation of primary macrophages and two-days PMA-differentiated THP-1 with dsDNA, ssDNA and poly (I:C) was conducted on 2 × 10^5^ cells in a 24-well format with 500 μl medium using lipofectamine 2000 (Life Technologies 11668-019) as carrier. Transfection protocols were as according to the manufacturer's instructions using a ratio of lipo-DNA/RNA of 1:1. For experimental details regarding concentration of DNA/RNA and time points before supernatant harvest and lyses of cells see figure legends.

### cGAMP stimulation of cells

Two-hundred thousand PMA-differentiated THP-1 cells in 24-well plates were permeabilized with digitonin permeabilization buffer (50 nM HEPES, 100 mM KCl, 3 mM MgCl_2_, 0.1 mM DTT, 85 mM sucrose, 0.2% BSA, 1 mM ATP, 1 mM GTP, pH 7) containing 5 μg ml^−1^ digitonin in the presence or absence of cGAMP. After incubation at 37 °C for 10 min, the permeabilization buffer was removed and replaced with warm RPMI medium with 10% FCS and 600 μg ml^−1^ glutamine. For experimental details regarding concentration of cGAMP and time points before supernatant harvest and lyses of cells see figure legends.

### Functional type I interferon assay

To quantify functional type I interferon the reporter cell line HEK-Blue IFN-α/β (InvivoGen) was utilized according to the manufacturer's instructions. Thirty thousand HEK-Blue cells were seeded in a 96-well plates with 150 μl medium devoid of Blasticidin and Zeocin and given 50 μl supernatant the next day. This cell line expresses secreted embryonic alkaline phosphatase under the control of the IFN- α/β inducible ISG54 promotor. SEAP activity was assessed by measuring optical density (OD) at 620 nm on a micro plate reader (ELx808, BioTEK). The standard range was made with IFN-α (A2) (PBL Assay Science).

### Enzyme-linked immunosorbent assay

Protein levels of the cytokines CXCL10 and TNF-α in supernatants, were measured using ELISA kits from PeproTech (CXCL10; 900-T39) and BioLegend (TNF-α; 430201) following the manufacturerŕs instructions.

### RNA analysis

Gene expression was determined by real-time PCR, using TaqMan detection systems (Applied Biosciences). Genomic RNA from cells was collected using the High Pure RNA Isolation kit (Applied Bioscience) and RNA was quality controlled by Nanodrop spectrometry. RNA for human *ISG54* (HS00533665), *IFNβ* (Hs01077958_s1), *RNaseP* (ThermoFisher #4316844) and *DAG1* (HS00189308_M1) were analysed with premade TaqMan assays and the RNA-to-C_t_-1 kit following manufactures procedures (Applied Biosciences). The MX3005 (Stratagene) was used for PCR quantification.

### Western blot

Two hundred thousand cells were lysed in 150 ul Pierce RIPA buffer (Thermo Scientific) supplemented with 10 mM NaF, 1 × complete protease cocktail inhibitor (Roche), 0.2% SDS, 1 × XT Sample Buffer (Bio-Rad) and 1x XT Reducing Agent (Bio-Rad). Whole cell lysates were sonicated using a Biorupture (Diagenode) 5 min at high intensity and denatured at 95 °C for 5 min before loading on gel. Separation was done on 10% or 4–20% SDS–PAGE gel electrophoresis (Criterion TGX gels, Bio-Rad). Transfer onto poly-vinylidene difluoride membranes (Bio-Rad) was done using a Trans-Blot-Turbo transfer system. All western blots were incubated and washed with TBS supplemented with 0.05% Tween-20. The following specific antibodies were used with PVDF membranes blocked in 5% skim-milk (Sigma Aldrich 70166-500G) and 1% skim-milk in antibody solutions (all 1:1,000 dilution): anti-IFI16 (Santa Cruz sc-6050), anti-cGAS (Sigma HPA031700), anti-STING (Cell Signaling #13647) and anti-vinculin (Sigma Aldrich v9131). The following specific antibodies were used with PVDF membranes blocked in 5% BSA (Roche 10 739 086 001) and 1% BSA in antibody solutions (all 1:1,000 dilution): anti-STING Ser^366^ (a gift from Zhijian James Chen, UT southwestern Medical school, Texas), anti-phospho-TBK1 (Cell Signaling #4947s), anti-TBK1 (Cell Signaling cat not), anti-IRF-3 (Cell Signaling #3013), and anti-phospho-IRF3 (Cell Signaling #5483s). Secondary antibodies (dilution 1:15,000), peroxidase-conjugated F(abŕ)2 donkey anti-mouse IgG (H+L), peroxidase-conjugated Affinipure F(abŕ)2 donkey anti-rabbit IgG (H+L) and peroxidase conjugated F(abŕ)2 donkey anti-goat IgG (H+L), were purchased from Jackson Immuno Research. IRF3 western blotting was conducted on nuclear fractions according to the manufacturer's instructions (Subcellular Protein Fractionation Kit for Cultured Cells, Thermo Scientific) using anti-IRF3 (Santa Cruz sc-9082). All membranes were exposed using Clarity Western ECL Blotting Substrate. The levels of proteins were for some experiments quantified by densitometry (ImageJ) as specified in the figure. To verify phosphorylation events on proteins, whole cell lysates were pre-treated with 10 units of FastAP Thermosensitive Alkaline phosphatase according to manufaturer's protocol (Thermo Scientific).

### Semi-native WB STING dimerization assay

STING dimerization was assayed under semi-native conditions. Two hundred thousand cells were lysed in 150 μl Pierce RIPA buffer (Thermo Scientific) supplemented with 10 mM NaF, 1 × complete protease cocktail inhibitor (Roche), 1 × XT Sample Buffer (Bio-Rad). Whole cell lysates were sonicated using a Biorupture (Diagenode) 5 min at high intensity before loading on gel without heating. Separation was done on 4–20% SDS–PAGE gel electrophoresis (Criterion TGX gels, Bio-Rad) where each gel was run initially for 10 min at 70 V and subsequently for 45 min at 120 V. Transfer onto poly-vinylidene difluoride membranes (Bio-Rad) was done using a Trans-Blot-Turbo transfer system. The blots were incubated and washed with TBS supplemented with 0.05% Tween-20. The following specific antibodies (used in 1:1,000 dilution) were used on membranes blocked in 5% skim-milk (Sigma Aldrich 70166-500G) and 1% skim-milk antibody solutions: anti-STING (Cell Signaling #13647) and anti-vinculin (Sigma Aldrich v9131). Secondary antibodies, peroxidase-conjugated F(abŕ)2 donkey anti-mouse IgG (H+L) and peroxidase-conjugated Affinipure F(abŕ)2 donkey anti-rabbit IgG (H+L). Membranes were exposed using Clarity Western ECL Blotting Substrate.

### Confocal microscopy

For visualization of IFI16 following transfection with DNA or cGAMP infusion, 50,000 cells on 1.2 mm coverslips were fixed with 2% PFA for 15 min and permeabilized with 0.2% Triton X-100. Coverslips were stained with antibodies directed against IFI16 (C-18, Santa Cruz sc-6050), STING (R&D Systems, AF6516) or IRF3 (Cell Signaling 4302, s). Secondary antibodies included Alexa Fluor 488 Donkey-anti-rabbit, Alexa Fluor 568 Donkey-anti-sheep, and Alexa Fluor 488 Donkey-anti-goat (Molecular probes, A11002, A21099 and A11055, resp). Images were acquired using a Zeiss LSM 710 or LSM 780 confocal microscope using a 63 × 1.2 water lens. Images were handled using Zen 2011 (Zeiss) and ImageJ.

### RNAseq analysis

Total RNA was extracted from 3 × 10̂6 Control or IFI16 KO macrophages using the High Pure RNA Isolation kit (Applied Bioscience) kit. Library preparation and RNAseq was performed at BGI (Shenzhen, China). In short, the 12 RNA samples (each condition in triplicate) were DNase I treated, enriched for mRNA using the oligo(dT) magnetic beads, and fragmented into 130 bp fragments. First strand cDNA was performed using random hexamers followed by second strand cDNA synthesis. After purification, end-reparation, adaptor ligation, and size-selected using TAE-agarose gel, the fragments were enriched by PCR amplification, and sequenced on an Ion Proton platform. A minimum of 10 M clean reads was obtained from each sample. Using Partek Flow, the reads were aligned to human hg38 (whole genome) using STAR 2.4.1d. The mapped reads were annotated using RefSeq transcripts 2015-08-04. Differential expressed genes were analysed with Partek Gene Specific Analysis Algoritm. A list of induced genes after stimulation was obtained by filtering the gene list using the following thresholds (1) fold-change >10, (2) *P<*0.01 and (3) total reads >30.

### Co-immunoprecipitation

Ten million THP-1 macrophages grown in T75 flask with 15 ml medium were transfected with 4 μg ml^−1^ dsDNA using lipofectamine. Cells were harvested and resuspended in 500 ul Pierce Co-IP lysis buffer (Thermo Scientific) supplemented with 1x Complete Ultra (Roche) and NaF 10 mM. Cells were allowed to lyse at 4C for 90 min under rotation and cytosolic supernatants were cleared by centrifugation at 2,000 *g* for 10 min. These lysates were then incubated at 4 °C overnight with 6 ug anti-IFI16 (Santa Cruz sc-6050) or 6 μg anti-STING (Cell Signaling 13647) primary antibodies. On the following day, each lysate was incubated with pre-washed Dynabeads magnetic protein G (Invitrogen), washed four times in Pierce Co-IP lysis buffer and proteins were detached from beads by incubating samples in a pH 2 elution buffer for 10 min on ice. Samples were subsequently neutralized, mixed with SDS-loading buffer and reducing agent, heated and loaded on SDS–Page.

### Liquid chromatography tandem mass spectrometry (LC-MS/MS)

Three million THP-1 cells were seeded in 6-well plates with 3 ml medium and allowed to PMA-differentiate for two days before transfecting with 2 μg ml^−1^ dsDNA. Cells were subsequently lyzed in (80% methanol, 2% acetic acid, 18% deionized water). Lysates were spun at 10.000 × g for five minutes before collecting supernatant A. The pellet was resuspended in 2% acetic acid and incubated for five minutes on ice before being spun at 10.000 × g. Supernatant B was collected and pooled with supernatant A. The previous step was repeated generating supernatant C which was pooled with A+B. Supernatants were added to HyperSep aminopropyl solid phase extraction (SPE) cartridges (Thermo Scientific) for cGAMP purification. The SPE cartridges were conditioned with methanol following deionized water before applying supernatants. After supernatant run-through columns were washed in deionized water followed by methanol. cGAMP was eluted in 1.5 ml alkaline methanol (80% methanol+20% concentrated aqueous ammonia (25% NH_4_OH)). Eluates were evaporated using a vacuum centrifuge and redissolved in 50 μl mobile phase A (0.1% aqueous formic acid).

The liquid chromatography system was a Waters Acquity UPLC system that consisted of a binary pump, a flow-through-needle sample manager thermostated at 5±2 °C and a column oven set at 45±2 °C (Waters). The tandem mass spectrometer was a Waters Xevo TQ-S triple-quadrupole instrument with an electrospray ionization (ESI) source. A volume of 10 μl of the purified cGAMP was injected onto a HSS T3 column (1.8 μm, 200 Å, 2.1 mm I.D. × 100 mm) (Waters) running 100% mobile phase A. The mobile phase was changed through a linear gradient to 80% A and 20% B (0.1% FA in acetonitrile) over 5 min. Then, the gradient was changed to 100% B over 0.1 min. Eight minutes after injection, the gradient was returned to 100% A over 0.1 min, and the column was equilibrated for 3.9 min before the next injection, resulting in a total runtime of 12 min. The column flow rate was 400 μl min^−1^. The source and desolvation temperatures were set at 150 °C and 600 °C, respectively, and the cone and desolvation nitrogen gas flows were set at 150 l h^−1^ and 1,000 l h^−1^, respectively. The mass spectrometer was operated in both positive and negative ion modes with a capillary voltage of 2.3 kV and a cone voltage of 30 V. The dominant precursor ions were the double charged molecular ions, *m/z* 338 in ESI(+) and *m/z* 336 in ESI(−). Several useful transition products were obtained by collision-induced dissociation using argon as collision gas. The transition products measured in ESI(+) were *m/z* 152 (obtained by applying a collision energy (CE) of 15 eV), *m/z* 524 (CE 9 eV) and *m/z* 136 (CE 22 eV). In ESI(−) *m/z* 134 (CE 18 eV) and *m/z* 150 (CE 18 eV) were measured. To achieve semi-quantitative results corresponding blank samples were spiked with cGAMP to a concentration of 10 nM (for single point calibration curves) or concentrations of 0.1, 1, 10, 100, 200, 300 and 400 nM (for 7-point calibration curves). The *m/z* 152 product ion obtained in ESI(+) was used as the primary quantifier.

### siRNA mediated knock down

On days 6 and 8 post isolation, monocyte derived macrophages were transfected with a pool of IFI16 specific siRNAs (#HSS105205,6,7; Life Technologies or #L-020004-00; Dharmacon) or the respective scrambled siRNA controls (45 nM) using Lipofectamine RNAiMax (Life technologies) according to the manufacturer's instructions, followed by infection or stimulation at day nine or day 10 respectively.

### Luciferase assay

HEK293T overexpressing STING cells were seeded in 6-well plates at a density of 5 × 10^5^ cells per well and cultured for 24 h. The cells were transiently transfected with a transfection mixture consisting of DNA and the transfection agent polyethylenimine-max (PEI-max) in a ratio of 1:3. For all experiments, the DNA mixture contained 968.5 ng reporter plasmid containing firefly luciferase under the control of the IFN-β promoter, 31.2 ng reporter plasmid containing Renilla luciferase under the control of the β-actin promoter and 1,000 ng plasmid DNA of the IFI16-wt, IFI16-PYRIN, IFI16-HIN-A type, cGAS, or eGFP as a control. The DNA and PEI mixtures were mixed and incubated 15 min at room temperature before applied to the cells. Cells were incubated for 18 h and reseeded on 96-well plate coated with poly-L-Lysine (Sigma 3438-100-01) and incubated for 18 h before infusion with cGAMP or digitonin buffer as a control for 10 min. After incubation media was removed and cells were lyzed (Promega E2920) and luciferase and Renilla signal was measured according to manufactory instructions.

### Sequencing

Genomic DNA was extracted and purified from THP1 KO cells using DNeasy Blood & Tissue Kit (Qiagen) followed by PCR amplification with primers designed to cover an area of 350 nucleotide around the gRNA target sequence in the IFI16 gene. PCR fragments were inserted into the TOPO-TA plasmid following the manufactures procedure. At least 10 individual clones from each gRNA target were evaluated by Sanger sequencing (GATC, Germany).

### Statistical analysis

For analysis of statistically significant differences between multiple groups of data we used unpaired Student *t*-test for multiple comparisons using Holm-Sidak. For analysis of three groups of data we used one-way ANOVA with Dunnett's multiple comparisons test.

### Data availability

Sequence data that support the findings of this study have been deposited in ENA with the primary accession code PRJEB18609. The data that support the findings of this study are available from the corresponding author on reasonable request.

## Additional information

**How to cite this article:** Jønsson, K. L. *et al*. IFI16 is required for DNA sensing in human macrophages by promoting production and function of cGAMP. *Nat. Commun.* **8,** 14391 doi: 10.1038/ncomms14391 (2017).

**Publisher's note:** Springer Nature remains neutral with regard to jurisdictional claims in published maps and institutional affiliations.

## Supplementary Material

Supplementary Information Supplementary Figures
