# Supplementary material for: IFI16 is required for DNA sensing in human macrophages by promoting production and function of cGAMP
Source: Nat Commun. 2017 Feb 10;8:14391. doi: 10.1038/ncomms14391 (PMC5309897; doi:10.1038/ncomms14391)

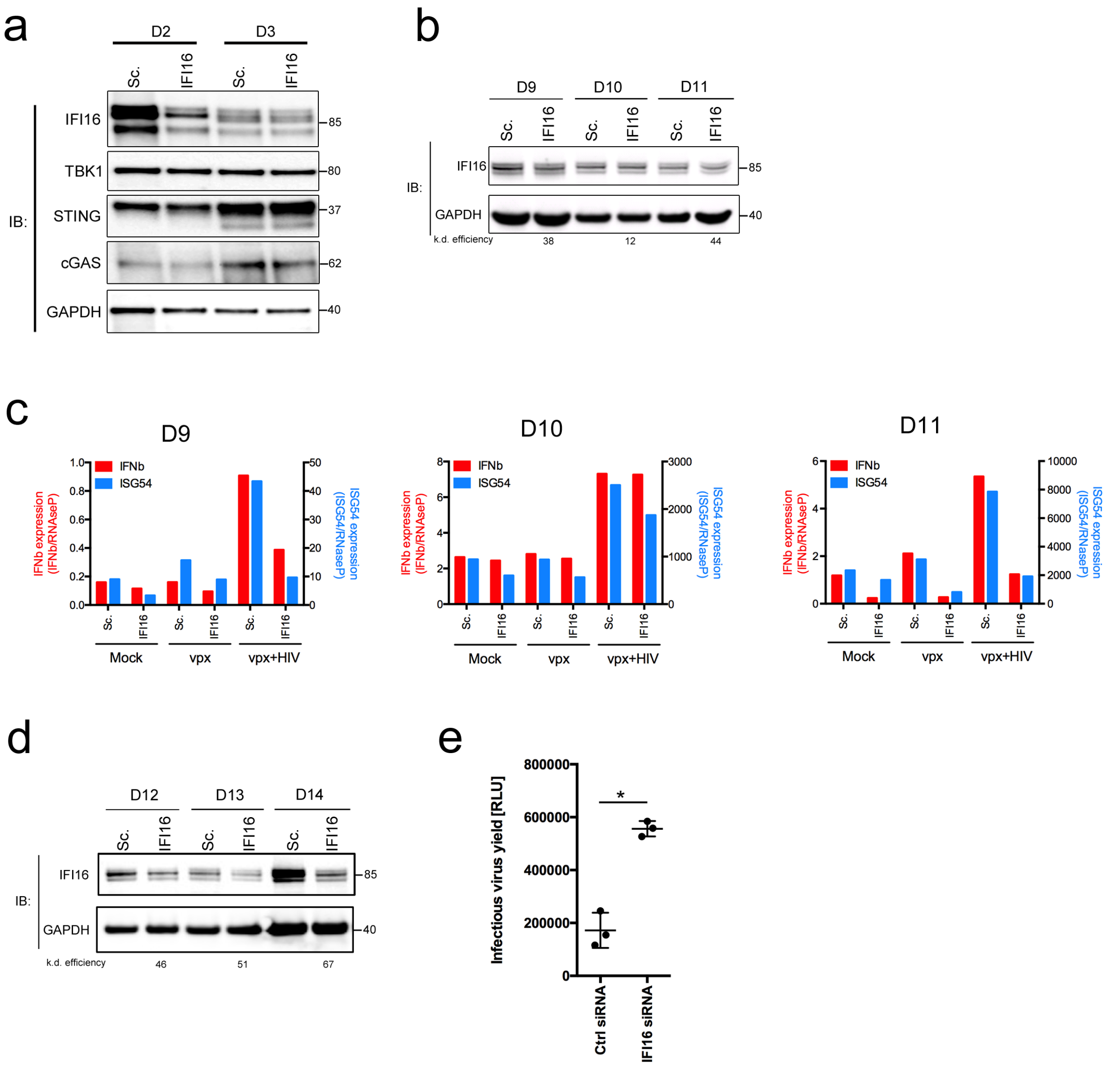

Supplementary Figure 1. Knockdown of IFI16 in MDMs with a secondary siRNA pool. (**a**) A detailed immunoblotting of donor 2 and 3 from Figure 1 demonstrating basal expression of TBK1, STING and cGAS. (**b+d**) Level of IFI16 expression was measured by immunoblotting in different MDMs donors treated with scramble (Sc.) and a second IFI16-specific (IFI16) siRNA pool. (**c**) *ISG54* expression and *IFN-β* expression was measured in the three donors portrayed in (**b**) challenged with either Vpx particles alone or HIV^{vpx+} for 18 hrs. Data represents mRNA expression of each gene normalized to mRNA expression of *RNaseP* from two biological replicates of each donor. (**d+e**) HIV replication was measured in the MDMs with a second IFI16-specific (IFI16) siRNA pool, 6 days post infection with HIV Bal.

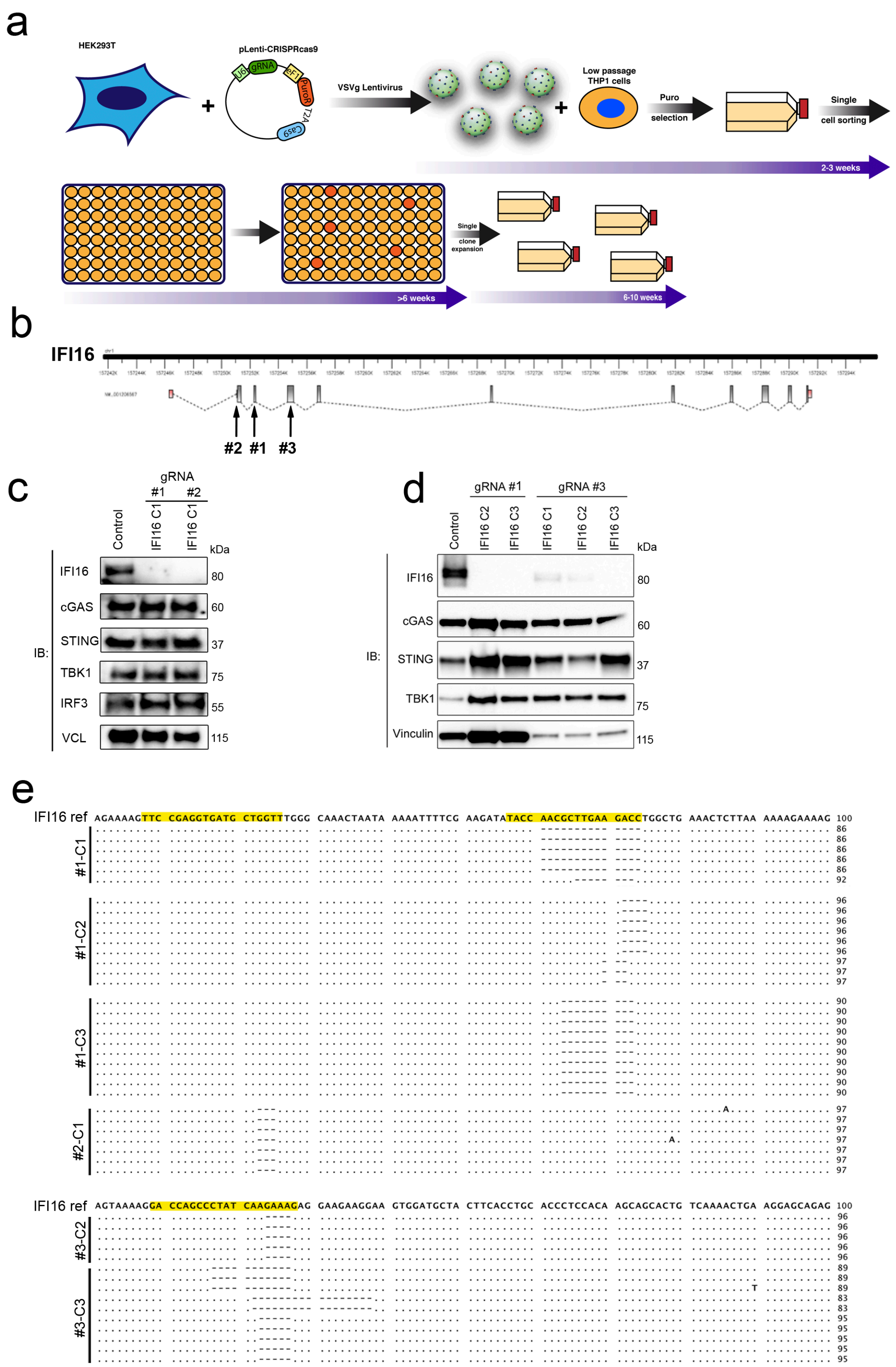

Supplementary Figure 2. Generation of CRISPR-Cas9 mediated gene knock out in THP-1 cells. (**a**) Schematic illustrating the workflow of generating specific gene knockout in THP-1 cells using the CRISPR-Cas9 technology. (**b**) Graphical representation of the specific gRNA targets for IFI16 using fancyGENE software analysis tool. Introns (dashed) and exons (grey). Black arrows

indicate Cas9 endonuclease mediated double stranded breaks. For information about the sequences see Materials and methods. (**c**) Effect of CRISPR gene disruption was evaluated by western blotting on PMA-differentiated THP-1 cells with the indicated immunoblotting (IB) for gRNA target 1 clone 1 and gRNA target 2 clone 1. (**d**) Evaluation of additional two IFI16 KO clones from gRNA target 1 and three clones from the third gRNA target. (**e**) Sequencing evaluation of the gene disruption in each THP1 IFI16 KO clone represented in (c+d) with the exception of gRNA #3 clone 1, which was not depleted of IFI16 and therefore excluded for further analysis. Yellow boxes represent the target area of the gRNA's.

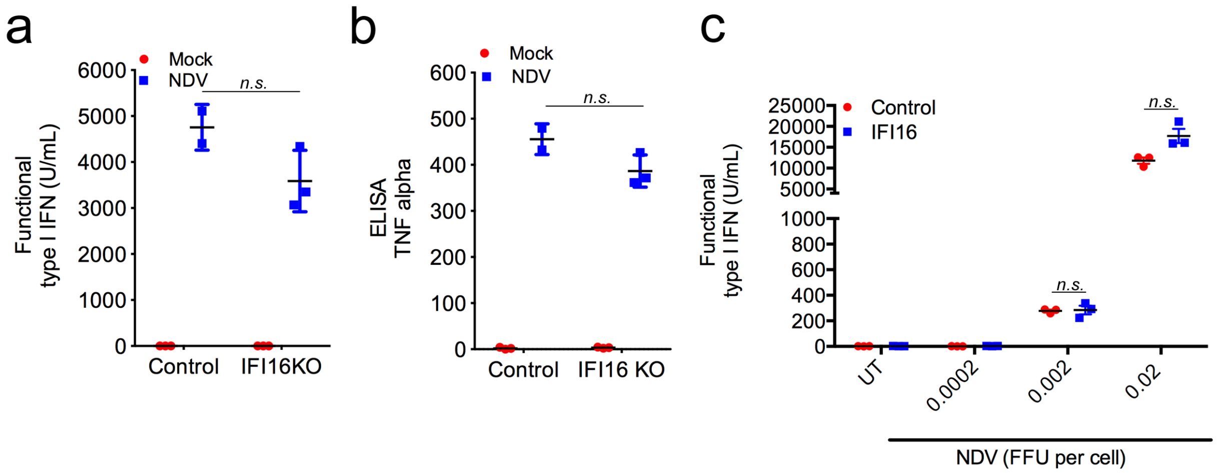

Supplementary Figure 3. Innate immune induction by NDV infection is independent of IFI16 expression. (**a**) Control and IFI16 KO cells were infected with NDV (FFU 0.01) for 20 hours and lysates evaluated for type I interferon expression using the HEK-Blue IFN-assay. (**b**) Same cell lysates from (**a**) were used to determine TNF-α expression using ELISA. (**c**). Control and IFI16 KO cells were infected with diluted series of NDV and type I interferon expression measured 20 hrs p.i. Data represent the mean ± SD of biological triplicates. Unpaired t-test was performed to evaluate the significance. *n.s., non-significant difference.*

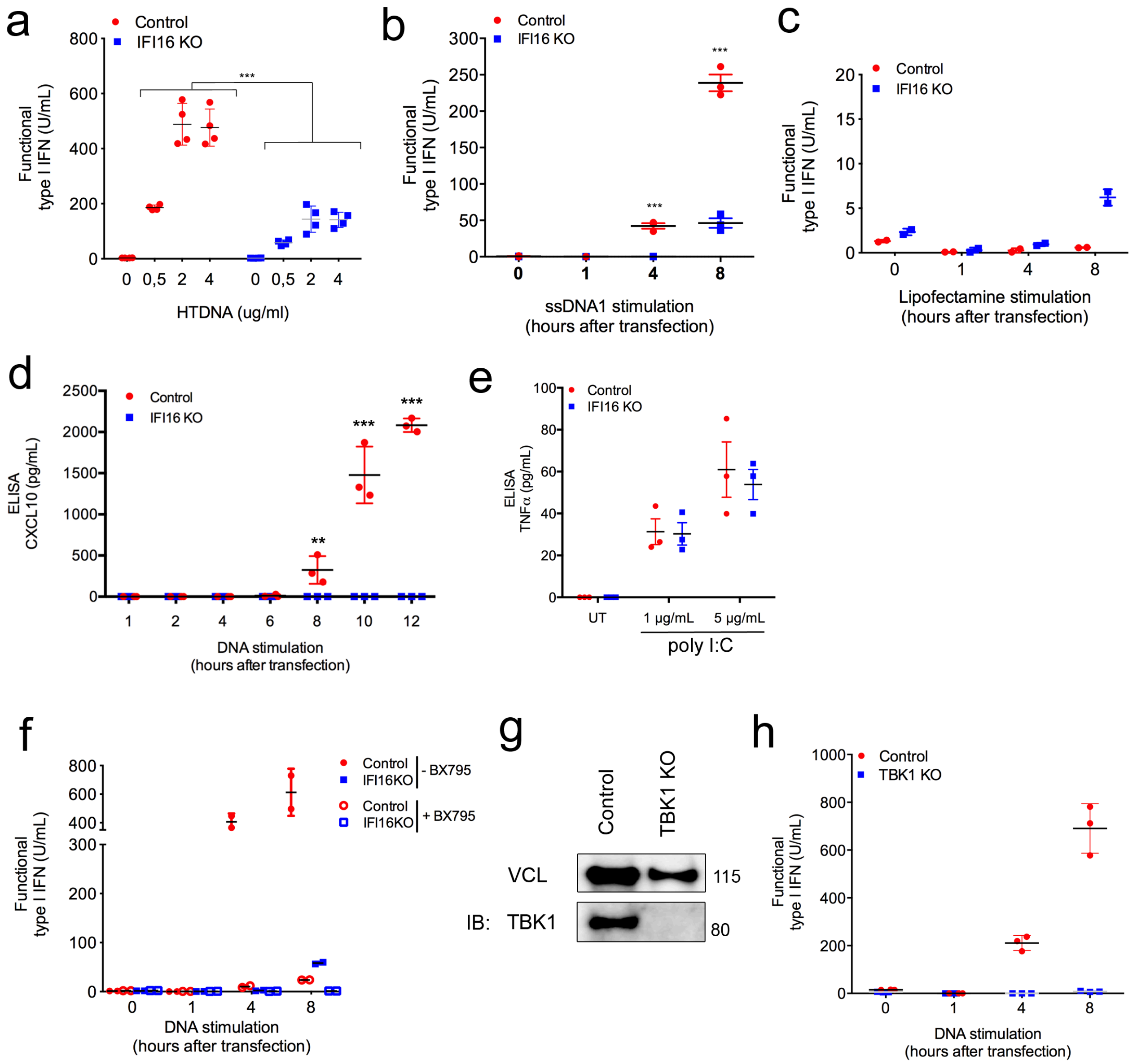

Supplementary Figure 4. Robust induction of type I interferon **by various forms of dsDNA is dependent on IFI16 expression.** Control and IFI16 KO THP-1 cells were stimulated by lipofectamine transfection using (**a**) Herring testis dsDNA (0.5, 2 or 4µg/ml) or (**b**) ssDNA1 (4µg/ml) and analysed for type I interferon induction. (**c**) As control of carrier, control and IFI16 KO cells were stimulated with lipofectamine (4µl/ml) and evaluated as in (**a** or **b**). (**d**) Control and IFI16 KO cells were stimulated with dsDNA (4µg/ml) at indicated time points and CXCL10 secretion measured by ELISA. (**e**) TNF-α ELISA analysis on supernatants from control and IFI16 KO cells stimulated with Poly I:C at indicated concentrations for 18 hrs. (**f**) Control and IFI16 KO cells were incubated with TBK1 inhibitor BX795 for 2 hrs prior to dsDNA transfection (4µg/ml).

Type I interferon secretion was measured at the indicated time points. (**g**) Immunoblotting of TBK1 in control and TBK1 KO cells. (**h**) Control and TBK1 KO cells were stimulated dsDNA (4µg/ml) and analysed for type I interferon induction at indicated time points.

Data represent the mean ± SD of biological triplicates, representative of three independent experiments. Unpaired t-test corrected for multiple comparisons using Holm-Sidak was performed to evaluate the significance. **P <0.05; ** P <0.01; *** P <0.001.*

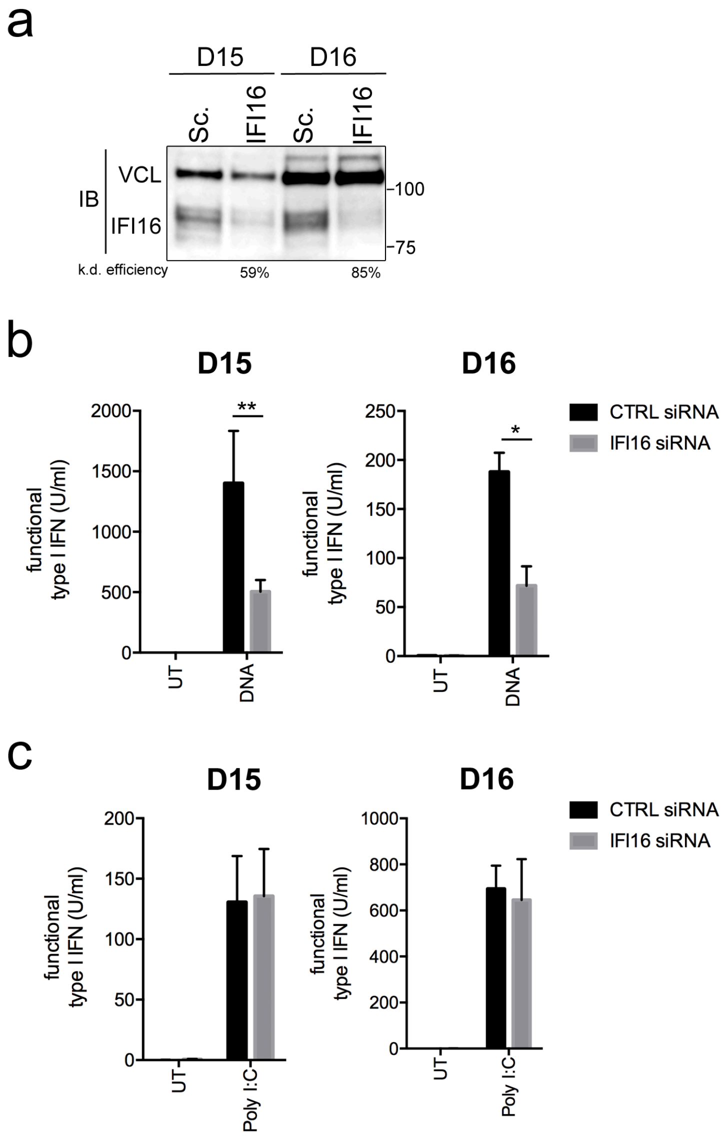

Supplementary Figure 5. Type I interferon **by dsDNA is dependent on IFI16 expression in primary human MDMs.** (**a**) Level of IFI16 expression was measured by immunoblotting in three MDMs donors treated with scramble (Sc.) and IFI16-specific (IFI16) siRNA pool. Donor 15 and 16 with significant IFI16 knockdown were stimulated with either (**b**) dsDNA (4ug/ml) or (**c**) poly(I:C) (1ug/ml) for 20 hrs and then analysed for type I interferon expression using the HEK-Blue IFN-bioassay. The donor 17 was excluded due to limited knockdown efficiency.

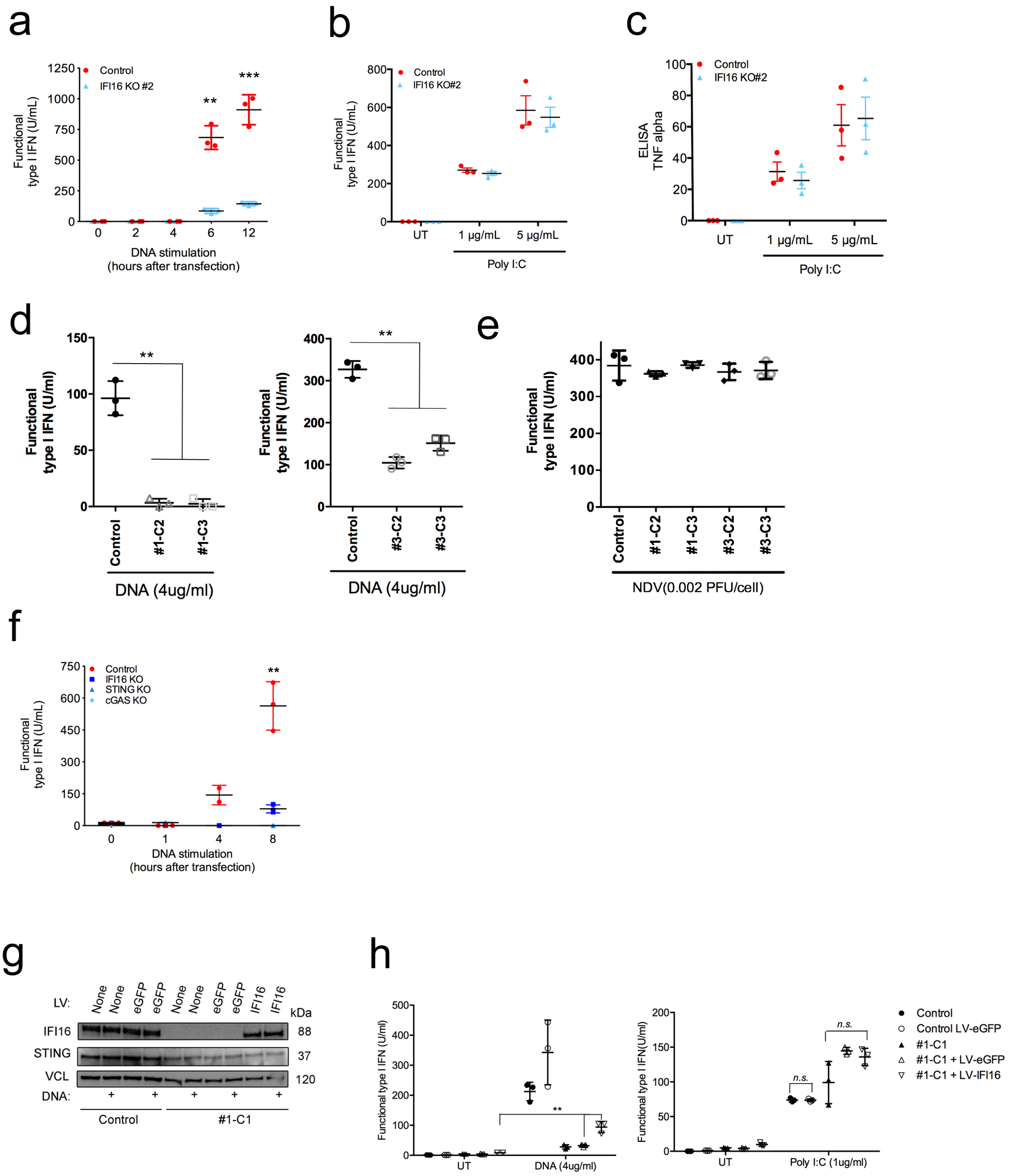

Supplementary Figure 6. Multiple CRISPR gRNA targeting IFI16 demonstrate similar phenotypes. Control and IFI16 KO #2 cells were stimulated with dsDNA (4µg/ml) at indicated time-points and evaluated for type I interferon induction (**a**); polyI:C (1µg/ml or 5µg/ml) for 18 hours and evaluated for type I interferon induction (**b**) or TNF-α protein expression (**c**). Four different PMA-differentiated THP-1 KO clones of IFI16 (see supplementary Figure 2d) were (**d**) transfected with dsDNA or (**e**) infected with NDV (0.002FFU/cell) for 20hrs and evaluated for type I interferon induction. (**f**) PMA-differentiated THP-1 cells from control, cGAS KO, STING KO and

IFI16 KO #2 were transfected with dsDNA (4µg/ml) at indicated time-points and evaluated for type I interferon induction. (**g**) IFI16 gene expression was reconstituted in THP1 IFI16 KO clone using lentiviral delivery. Forty-eight hours later cells were transfected with (**h**) dsDNA (4ug/ml) or PolyI:C, and evaluated for type I interferon responses.

Data represent the mean ± SD of biological triplicates, representative of three independent experiments. Unpaired t-test was performed to evaluate the significance. *$^*P < 0.05$; $^{**}P < 0.01$.*

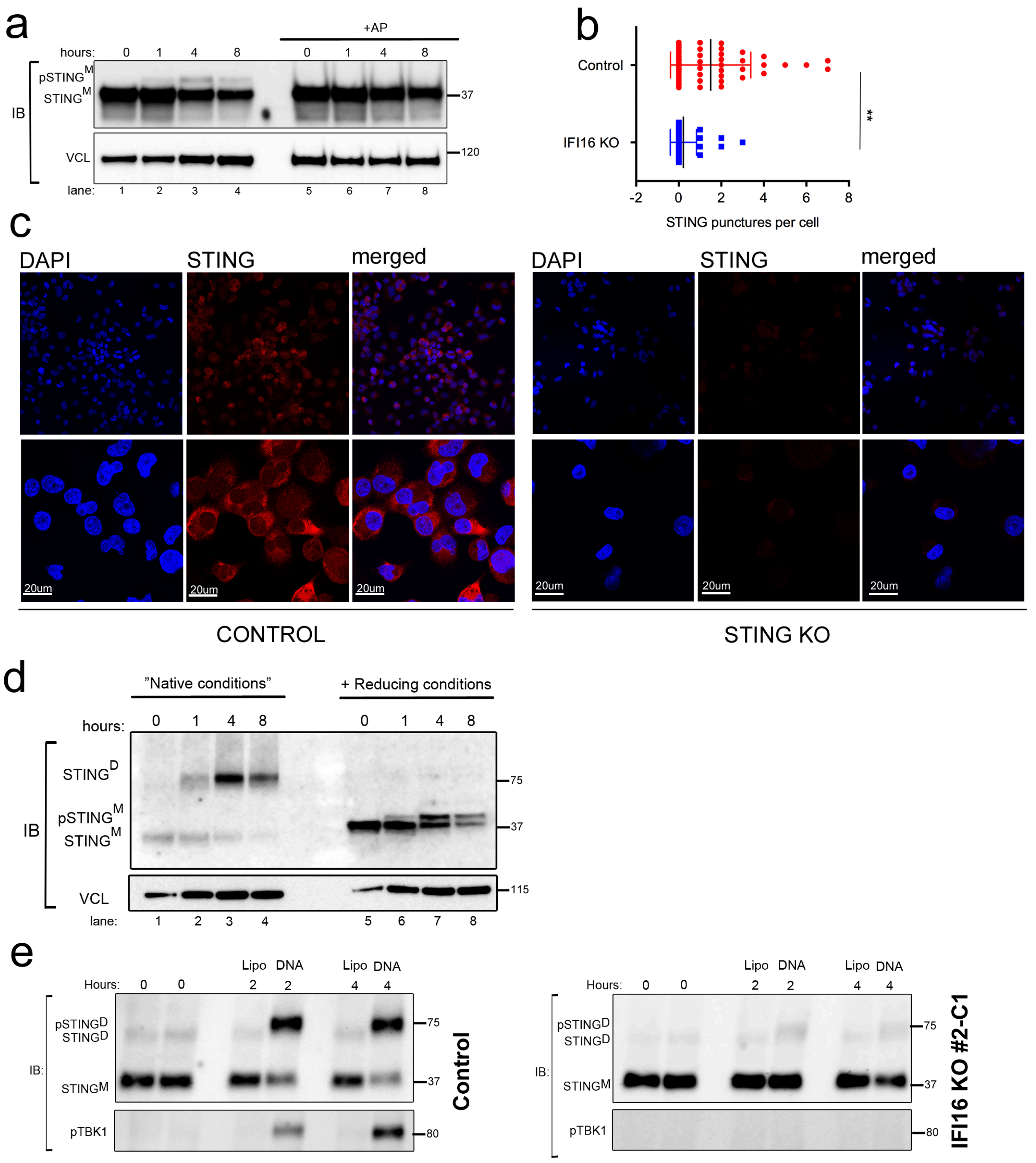

Supplementary Figure 7: STING dimerization upon DNA stimulation. (**a**) Whole cell lysate from control cells stimulated with dsDNA (4µg/ml) at indicated time-points were left untreated or treated with alkaline phosphatase for 30 minutes before SDS-Page gel electrophoresis and immunoblotting with antibodies against STING and vinculin (VCL). Data are representative of two independent experiments. (**b**) STING puncta were quantified by counting fifty separate images of control or IFI16 KO cells 4 hrs p.t. (corresponding to Figure 2e). (**c**) Confocal microscopy illustrating STING expression in THP1 Control or STING KO cells with (upper, x40; lower x63-olie objectives). (**d**) Control THP-1 cells were stimulated with dsDNA (4µg/ml) at indicated hours

and subjected to either native or non-native gel electrophoresis including reducing agents. Immunoblotting was done with antibodies against STING. Vinculin (VCL) was used as loading control. Data are representative of two independent experiments. (**e**) Control and IFI16 KO #2 THP-1 cells were stimulated with lipofectamine or lipofectamine+dsDNA (4µg/ml) at indicated hours and subjected to semi-native gel electrophoresis and immunoblotting with antibodies against STING and pTBK1. Data are representative of two independent experiments.

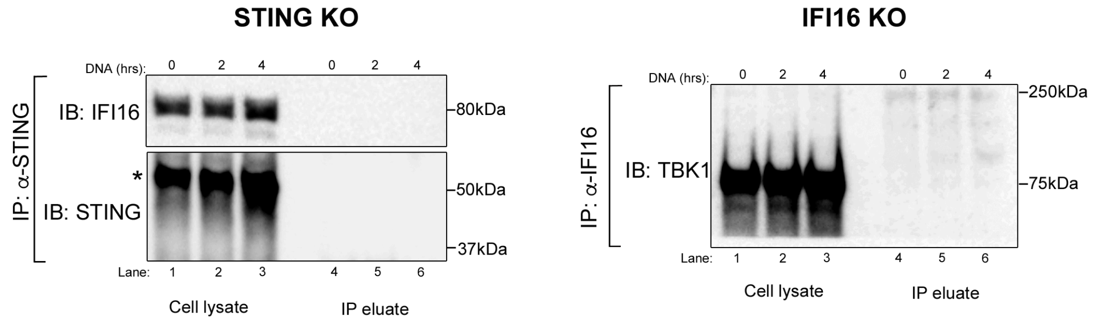

Supplementary Figure 8. Cleared cell lysates (CCL) of THP-1 STING KO or IFI16 KO cells stimulated with dsDNA (4µg/ml) for 2 and 4 hrs were subjected to over-night co-immunoprecipitation with antibodies indicated in each panel. Lysates from control cells were co-IP with STING (left panel) or IFI16 (right panel). Input and elutes were analysed by gel electrophoresis followed by immunoblotting (IB) with the indicated antibodies. Each blot is representative of two independent experiments. Asterisk marker indicates an unspecific band at approximately 50kDa in the cell lysate fraction. The specific band for STING is 37 kDa.

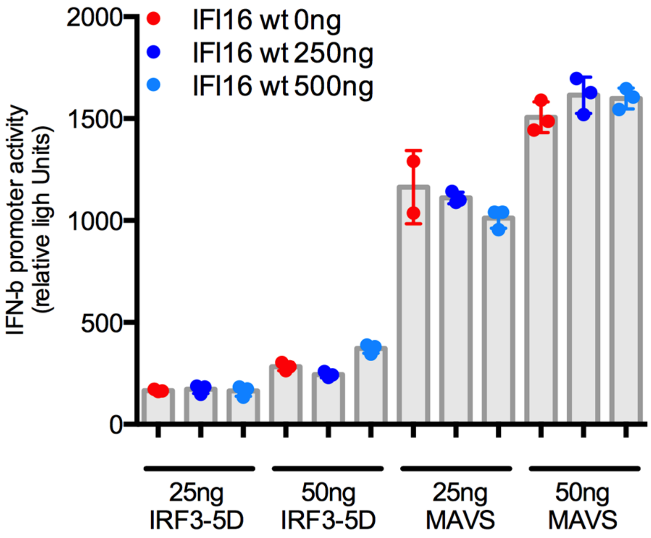

Supplementary Figure 9. IRF3 and MAVS function is not affected by IFI16. HEK293T cells were transfected with either 25ng or 50ng plasmid encoding for the constitutive active mutant of IRF3 (IRF3-5D) or MAVS, together with increasing doses of plasmids expressing IFI16 wildtype. Level of activation was evaluated 24 hrs later by measuring expression of an IFN-β promoter driven Firefly gene normalized to a beta-actin promotor Renilla gene. Values from empty vector control were extracted from each dataset. Data represent the mean ± SD of biological triplicates, representative of two independent experiments.

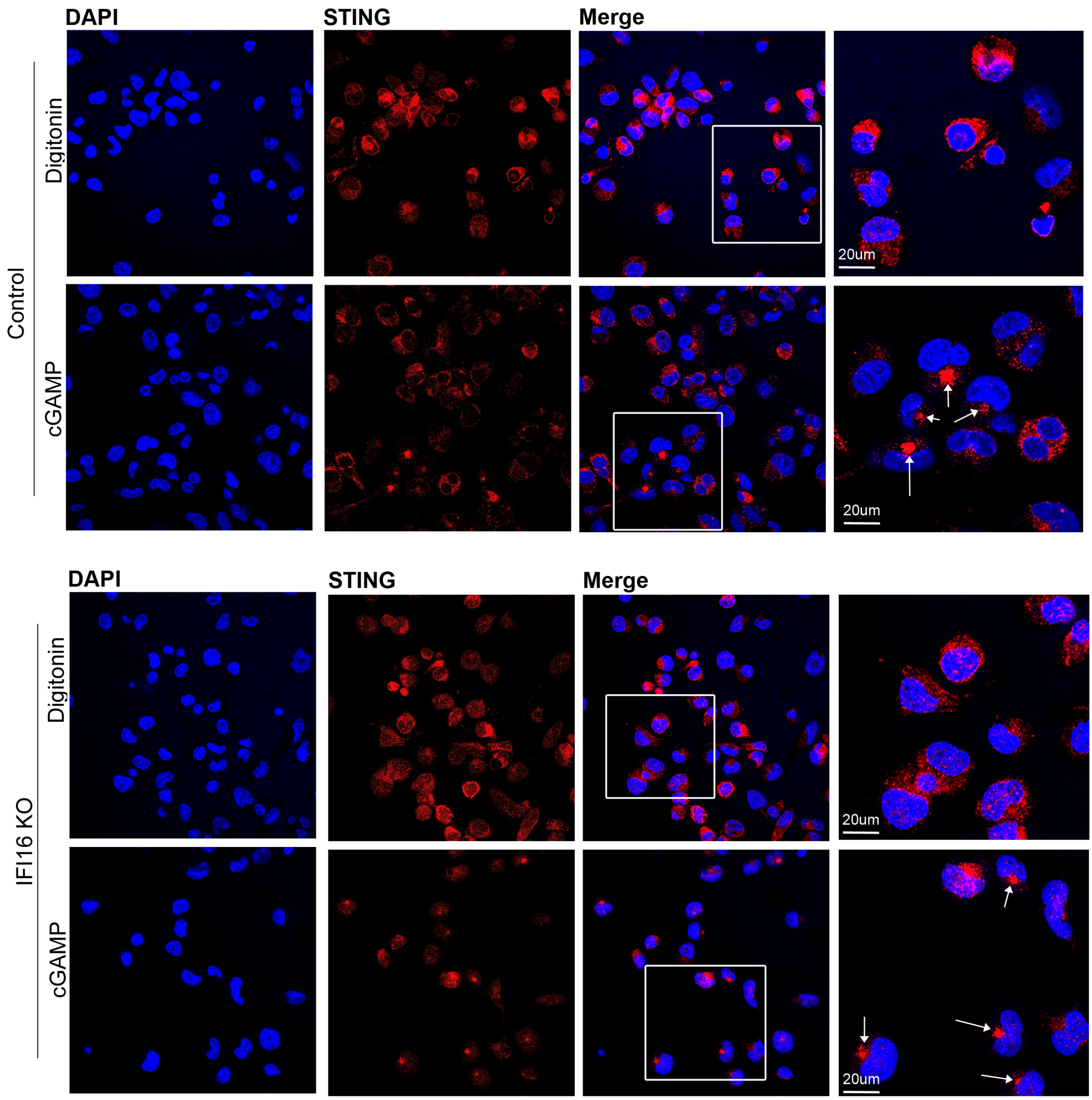

Supplementary Figure 10. STING foci formation following cGAMP stimulation. STING trafficking from ER localisation to cytosolic puncta was evaluated in control and IFI16 KO cells infused with 50nM cGAMP for 1 hour. Cells were fixed and stained for DAPI (blue) and STING (red). Arrows indicate the generation of STING foci formations.

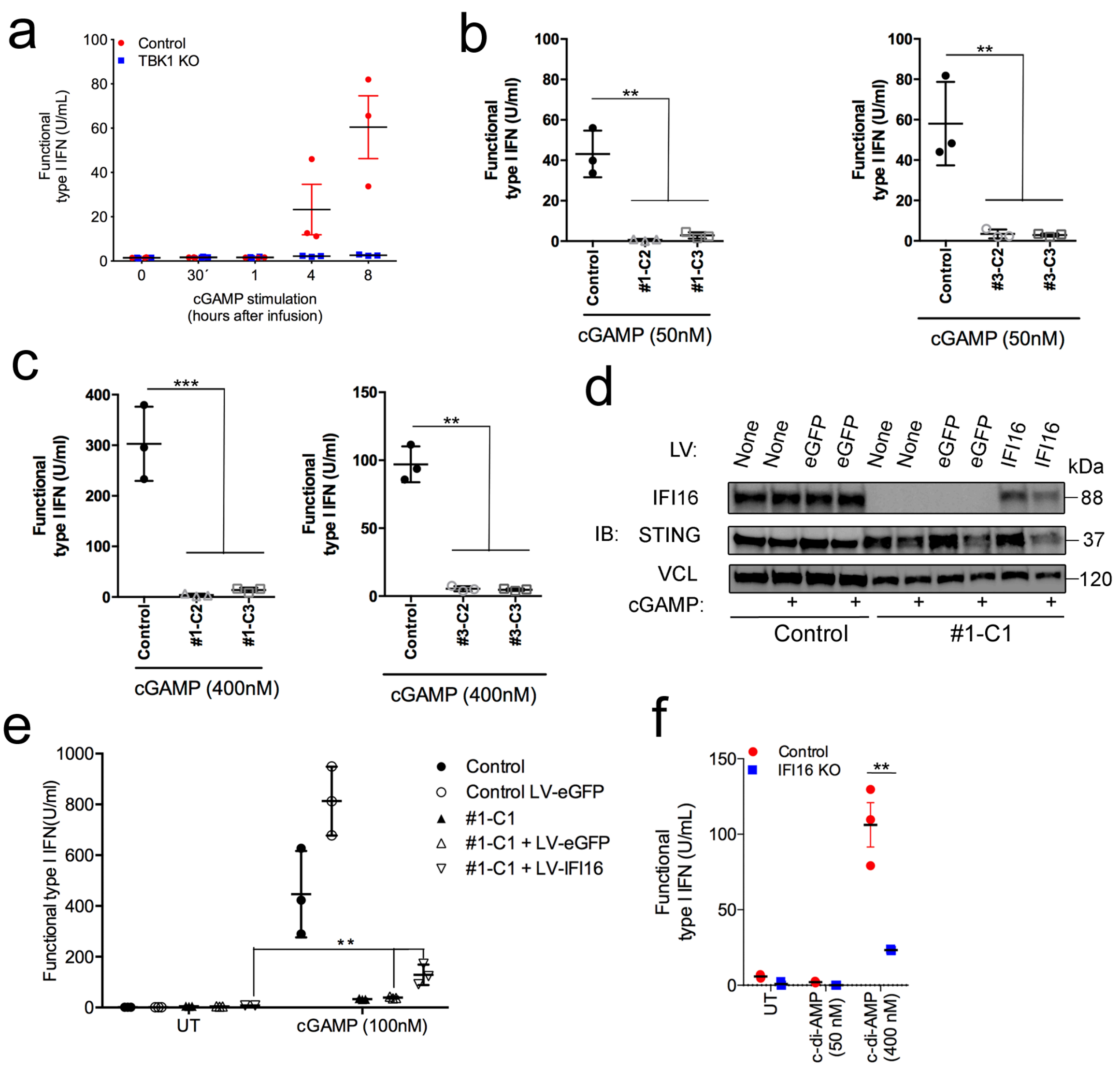

Supplementary Figure 11. (**a**) Control and TBK1 KO THP-1 cells were stimulated with cGAMP (50nM) and type I interferon secretion was evaluated at indicated time points. (**b+c**). Four different PMA-differentiated THP-1 KO clones of IFI16 (see supplementary Figure 2d) were infused with (**b**) 50nM or (**c**) 400nM cGAMP and evaluated for type I interferon induction 20 hrs later. (**d**) IFI16 gene expression was reconstituted in THP-1 IFI16 KO clone using lentiviral delivery. **(e)** Forty-eight hours later cells were infused with cGAMP (100nM) and evaluated for type I interferon responses after 8 hrs. (**f**) Control and IFI16 KO THP-1 cells were infused with low (50nM) and high doses (400nM) of cyclic-di-AMP (c-di-AMP) and evaluated for type I interferon induction 20 hrs later. Data represent the mean ± SD of biological triplicates, representative of three independent experiments. Unpaired t-test was performed to evaluate the significance. **P <0.05; ** P <0.01.*

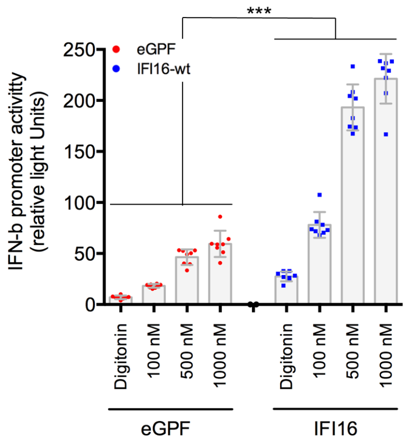

Supplementary Figure 12. HEK293T^{STING} cells were transfected with either control plasmid (eGFP) or IFI16-wt plasmid at 100ng/well. Twenty-fours later cells were stimulated with increasing doses of cGAMP infused with digitonin. STING activation was evaluated 24 hrs later by measuring expression of an IFN-b promoter Firefly gene normalized to a beta-actin promotor Renilla gene.

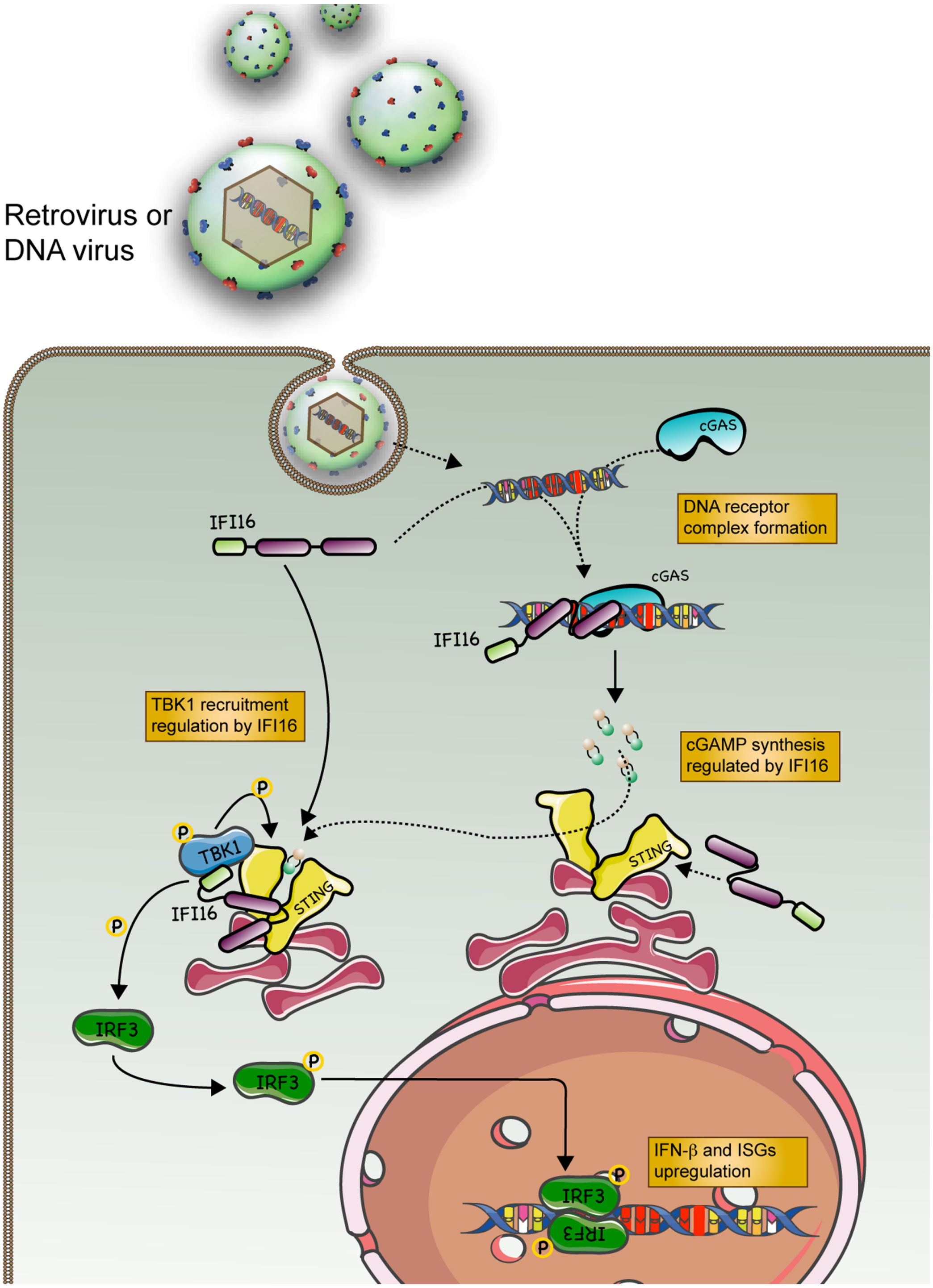

Supplementary Figure 13. Proposed two-step model of the function of IFI16 in regulating the STING signalling events following DNA sensing in human macrophages.

Supplementary figure 14 Full-Length Western Blots

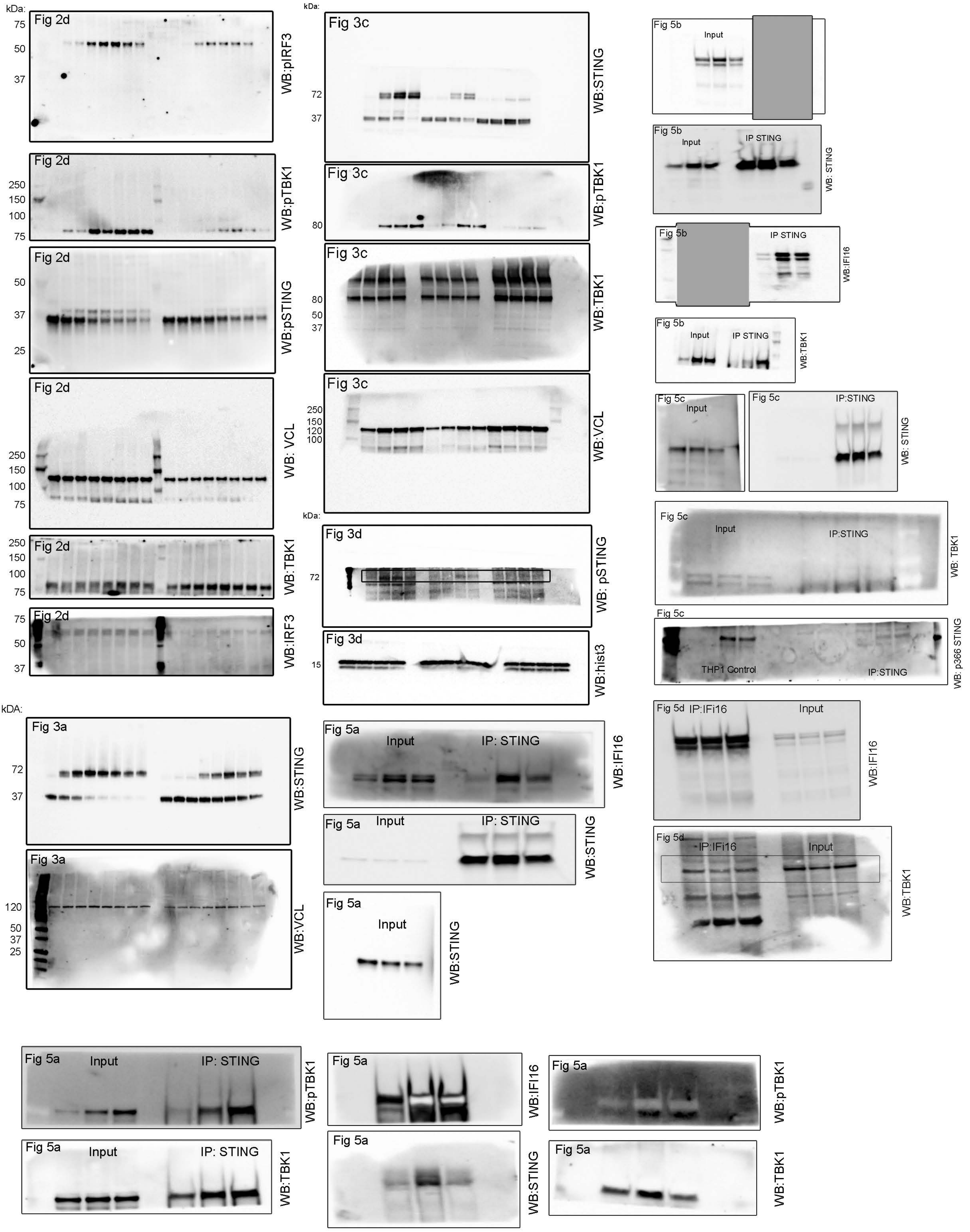

Supplementary figure 14, cont.

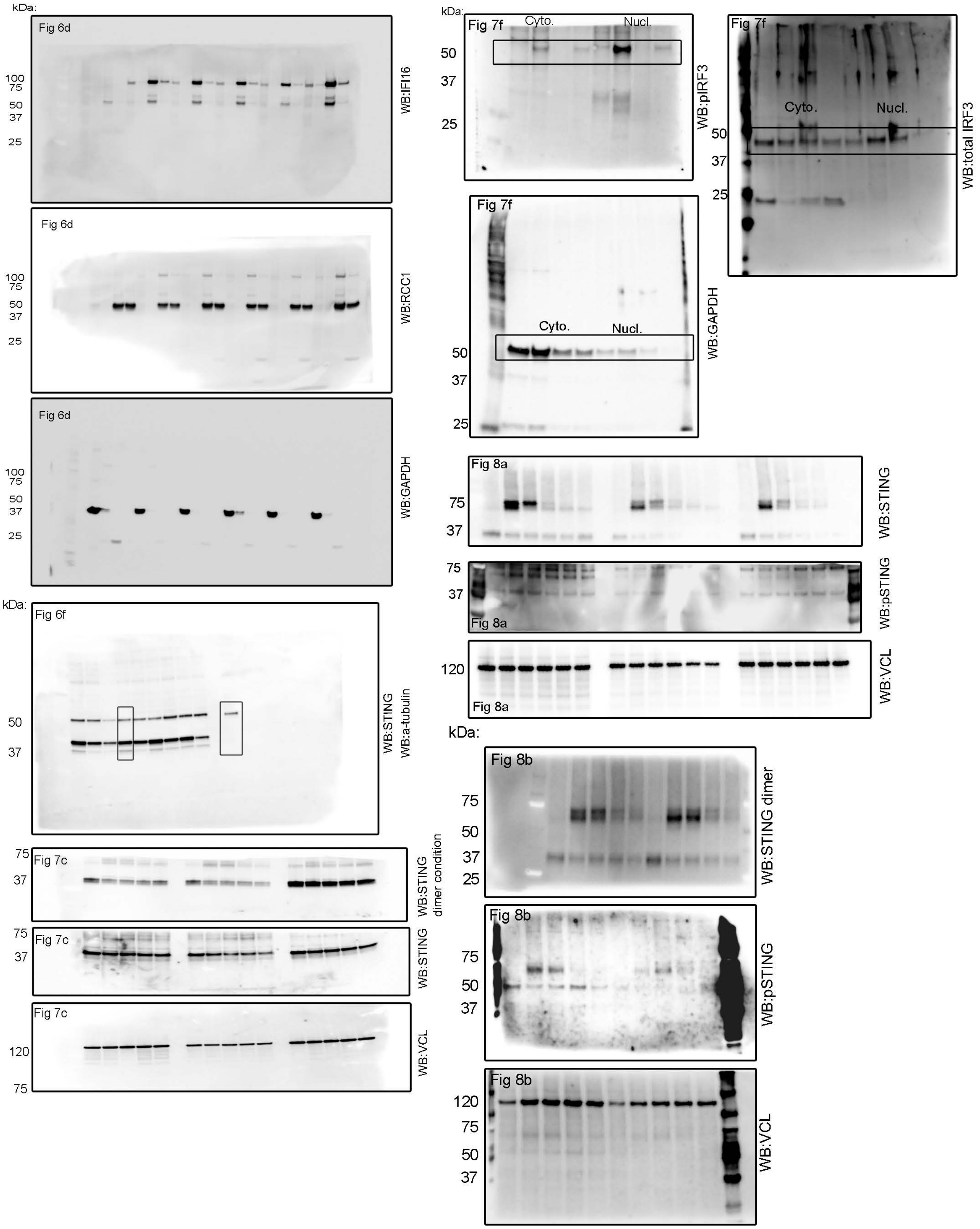

Supplementary figure 14, cont.

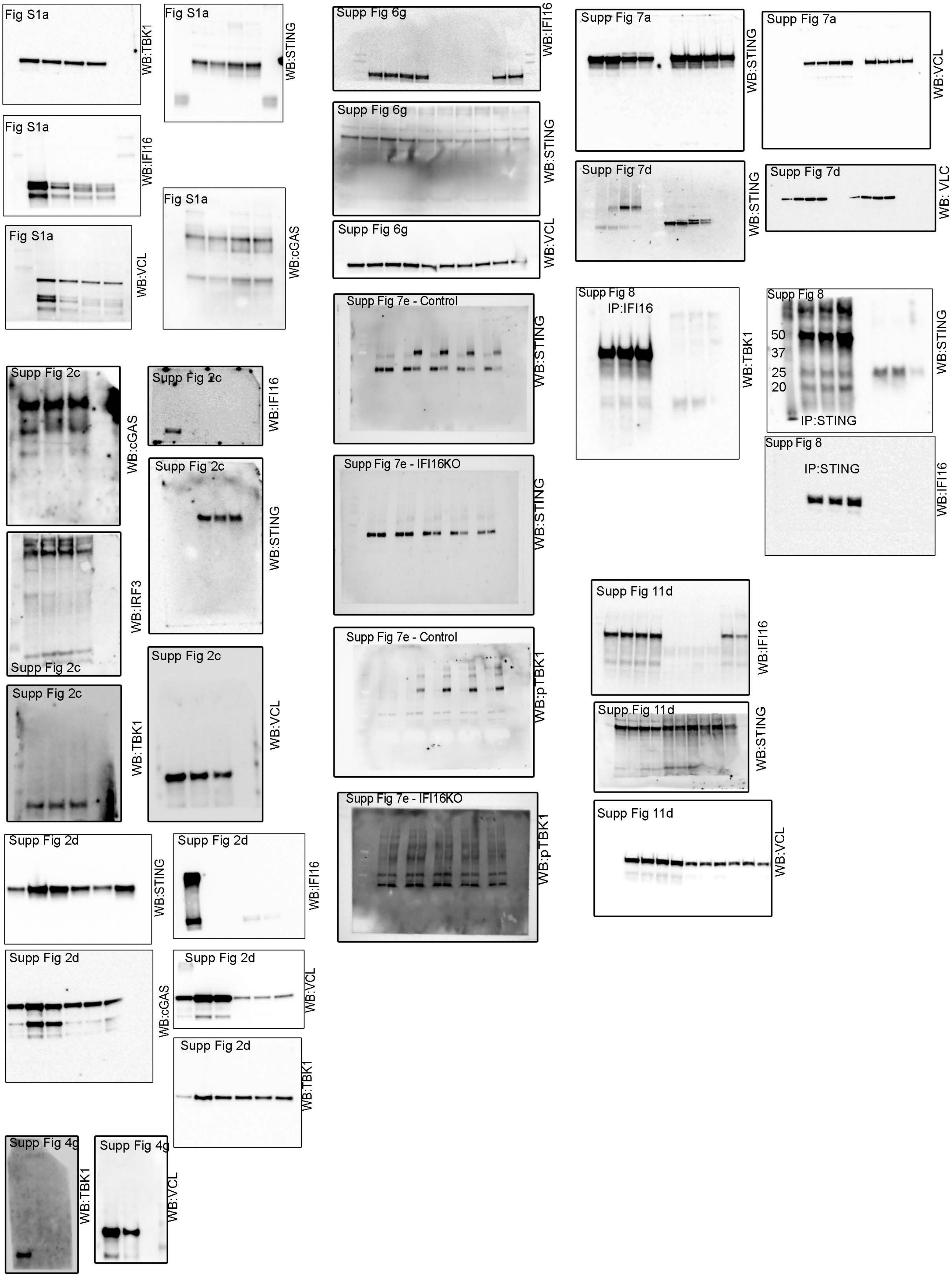

Supplement: Supplementary Information — Supplementary Figures [file ncomms14391-s1.pdf]
